# Bisphenol A: an emerging threat to female fertility

**DOI:** 10.1186/s12958-019-0558-8

**Published:** 2020-03-14

**Authors:** Claudia Pivonello, Giovanna Muscogiuri, Antonio Nardone, Francesco Garifalos, Donatella Paola Provvisiero, Nunzia Verde, Cristina de Angelis, Alessandro Conforti, Mariangela Piscopo, Renata Simona Auriemma, Annamaria Colao, Rosario Pivonello

**Affiliations:** 1 Dipartimento di Medicina Clinica e Chirurgia, Sezione di Endocrinologia, Università “Federico II” di Napoli, Via Sergio Pansini, 5, Naples, Italy; 2 Dipartimento di Sanità Pubblica, Università “Federico II” di Napoli, Naples, Italy; 3 FERTISEXCARES Centro di Andrologia, Medicina della Riproduzione e della Sessualità Maschile e Femminile, Università “Federico II” di Napoli, Naples, Italy; 4 I.O.S. & COLEMAN Srl, Naples, Italy; 5 Dipartimento di Neuroscienze, Scienze Riproduttive ed Odontostomatologiche, Università “Federico II” di Napoli, Naples, Italy; 6 Laboratory of Seminology-sperm bank “Loredana Gandini”, Department of Experimental Medicine, University of Rome “La Sapienza”, Rome, Italy; 7 Cattedra Unesco “Educazione alla salute e allo sviluppo sostenibile”, Università “Federico II” di Napoli, Naples, Italy

**Keywords:** BPA, Fertility, PCOS, Endometriosis, Obesity, Insulin resistance

## Abstract

Bisphenol-A (BPA) has been reported to be associated to female infertility. Indeed, BPA has been found to be more frequently detected in infertile women thus leading to hypothesize a possible effect of BPA on natural conception and spontaneous fecundity. In addition, in procedures of medically assisted reproduction BPA exposure has been found to be negatively associated with peak serum estradiol levels during gonadotropin stimulation, number of retrieved oocytes, number of normally fertilized oocytes and implantation. BPA deleterious effects are more critical during perinatal exposure, causing dysregulation of hypothalamic-pituitary-ovarian axis in pups and adults, with a precocious maturation of the axis through a damage of GnRH pulsatility, gonadotropin signaling and sex steroid hormone production. Further, BPA exposure during early lifestage may have a transgenerational effect predisposing the subsequent generations to the risk of developing BPA related disease. Experimental studies suggested that prenatal, perinatal and postnatal exposure to BPA can impair several steps of ovarian development, induce ovarian morphology rearrangement and impair ovarian function, particularly folliculogenesis, as well as can impair uterus morphology and function, in female adult animal and offspring. Finally, studies carried out in animal models have been reported the occurrence of endometriosis-like lesions after BPA exposure. Moreover, BPA exposure has been described to encourage the genesis of PCOS-like abnormalities through the impairment of the secretion of sex hormones affecting ovarian morphology and functions, particularly folliculogenesis. The current manuscript summarizes the evidence regarding the association between BPA exposure and female infertility, reviewing both clinical and preclinical studies.

## Introduction

In the last decades the environmental pollution caused by urbanization and industrialization has been reported to affect human health [1, 2]. Endocrine disrupting chemicals (EDCs), which can be found in agriculture, industry, drugs or food chain, comprise a wide variety of exogenous chemicals including synthetic compounds able to affect hormones synthesis, metabolism, and function [3]. The EDCs exposure can occur via ingestion of water, food and dust, via inhalation of gases and air particles and via dermal absorption of cosmetics and/or substances deriving from thermal paper. Moreover, a transmission from the pregnant woman to the developing fetus or child, during gestation and lactation, through the placenta and breast milk, was also demonstrated [4, 5]. After absorption, EDCs can accumulate in fat tissue for a long time and their metabolites can be detrimental to the human organism but it may not become evident until later in life [6]. Despite this evidence, the long-term effects of EDCs exposure were not deeply investigated. EDCs exert their actions triggering mainly genomic mechanisms but also promoting non-genomic actions [7–9], through the binding to several hormone receptors, including thyroid and, especially, steroid receptors, mainly estrogen (ER) or androgen (AR) receptors, compromising reproductive system and negatively affect the fetal and neonatal development and physiology [10]. Despite several evidences in literature on the potential effects of EDCs on human reproduction, the molecular mechanisms underlying these effects are not completely understood.

Bisphenol-A [2,2-bis(4-hydroxyphenyl) propane (BPA), CAS No. 80–05-7] is one of the most investigated EDCs. BPA is largely found in polycarbonate resin mainly used for plastic bags, bottles and packaging, particularly water and milk bottles, coated tins, particularly food and drink cans, and microwave ovenware [11–13]. The primary source of exposure to BPA is diet; indeed, BPA, being a constituent of food containers and packaging, can leach into food products, especially after heating [14, 15]. In humans, BPA is adsorbed by gastrointestinal tract, metabolized in the liver and finally excreted by urine [16]. Several studies have highlighted the negative effects of BPA on reproductive system [17]. Particularly, it has been shown that BPA displays a high affinity for ER, having an estrogen-mimicking behavior and consequently stimulating estrogen function [11–13]; therefore, BPA has been hypothesized to be involved in several diseases of female reproductive system [3, 18–22], due to its property to stimulate ER-dependent gene expression involved in the pathophysiology of female reproductive system [23–26]. Moreover, BPA is also able to inhibit androgen function by binding AR [27]. Particularly, BPA is able to influence ovarian morphology [6] and function, especially steroidogenesis [28–34] and folliculogenesis [32, 34–36], and, as well as uterine morphology [37] and function, especially uterine receptivity, consisting of the uterine ability to accommodate embryo attachment [38–40], and embryo implantation [37–40]. The impairment of endocrine function, and the consequent impairment of ovarian and uterine morphology and function, may lead to the development of several diseases involving reproductive system, especially ovary and uterus [19, 41, 42]. The ultimate consequence of the impairment of endocrine function and the morphology and function of the female reproductive system may be represented by female infertility.

The current review aims to give an overview of the link between BPA exposure and female infertility by reporting the few epidemiological studies available in humans and by providing, in particular, in vitro, ex vivo and in vivo animal-based evidences of BPA implication in the pathogenesis and progression of female infertility. In particular, studies describing the BPA effect observed on regulation of hypothalamus-pituitary-ovary (HPO) axis and on female reproductive organs (ovary, oviduct, uterus and vagina) morphology and functions (estrous cyclicity, steroidogenesis, folliculogenesis, uterine receptivity, embryo implantation and vaginal opening) will be focused. Moreover, the current manuscript will critically review the observational studies in humans evaluating the association of serum and/or urinary BPA levels and female infertility mostly focusing on natural conception, in medical assisted reproduction (MAR) outcome and on infertility–related reproductive disorders, particularly endometriosis and polycystic ovary syndrome (PCOS).

## Search strategies

Articles were individually retrieved by each author up until February 2018, by search in PubMed (MEDLINE) using the following search terms: ‘bisphenol- A’, ‘ovary’, ‘uterus’, ‘HPO axis’, ‘oviduct’, ‘vagina’, ‘PCOS’, ‘endometriosis’, ‘endocrine disruptor’, ‘female fertility’, ‘female infertility’, ‘time to pregnancy’, ‘environment’, ‘endocrine disruptors’. The reference lists of relevant articles and reviews were also searched manually.

## Definition of BPA doses and exposure

The proper definition of BPA low-doses or high-doses range has been extensively discussed. In the current review, in accordance with the Chapel Hill BPA expert panel consensus statement [43], “low BPA doses” have been considered as follow: 1) in human epidemiological studies, doses below the reference dose of Tolerable Daily Intake (TDI), corresponding to 0.05 mg/kg (50 μg/kg) body weight/day (bw/day) that, as established by the United States Environmental Protection Agency (EPA), is based, on grounds of prudence, on a 1000-fold reduction of lowest observed adverse effect level (LOAEL), defined by US National Toxicology Program (NTP) and corresponding to 50 mg/kg/day for oral exposure in laboratory animals in traditional toxicological studies conducted for risk assessment [43, 44]; 2) in animal models, doses below the LOAEL (50 mg/kg bw/day) [43, 44] and 3) in in vitro models, doses below 1 × 10^−7^M for cell culture experiments, corresponding circulating BPA levels in animals after administration of BPA at LOAEL concentration [44]. BPA levels higher than that previously specified have been considered “high BPA doses”. In experimental in vivo studies, BPA exposure is finalized to observe BPA effects in female adult animals or in both female adult pregnant animals and in female offspring. Most of the studies have been performed on laboratory mouse and rat models with variation regarding to the exact timing of exposure during development. BPA exposure of female offspring have been considered “prenatal” when female adult pregnant animals have been treated and consequently female fetuses have been exposed from the embryonic day 1 (E1) to E17; “perinatal” when female adult pregnant animals have been treated and female fetuses have been exposed from E18 until the day of birth; and “postnatal” when female pups have been exposed after birth from PND1 to approximately 3 weeks of age (PND21-PND24). BPA exposure have been also evaluated on female pups in pre-pubertal and pubertal phases till the animals reach sexual maturity (from PND25 to PND47,) and on female adult animals (starting from PND48) [45].

## Bisphenol a as endocrine disruptor chemical: mechanisms of action

BPA is a chemical mainly used as a monomer in the manufacturing of polymers, in particular polycarbonate resins, but also epoxy polyester, polysulfone and polyacrylate resins, and flame retardants [11–13]. Since the polycarbonate is used for the production of plastic bags, bottles and packaging, particularly water and milk bottles and including infant feeding bottles, coated tins, particularly food and drink cans, and microwave ovenware, BPA exposure is essentially conveyed via diet, contributing to more than 90% of the overall exposure [15]. BPA may leach into food depending on temperature (high temperature) and pH (alkaline conditions) [15]. BPA may reach the body by ingestion and or absorption of materials derived from leaching of resin-based dental filling products [15]. BPA is also used to print surface of thermal paper like airlines and train tickets as a heat-activated developer; therefore, the handling of thermal paper represent a different way to BPA exposure, which occur via dermal absorption, similarly to the exposure induced by the cosmetics [46]. The exposure to BPA occurring through the dermal absorption by the handling of thermal paper or by the application of cosmetics, together with air inhalation and dust and dental material ingestion, represents only the 5% of alternative and less common exposure [15].

BPA daily human exposure, assessed by measuring the urinary excretion of BPA, may vary widely worldwide, as well as the daily dietary intake, assessed by measuring BPA levels in food [13]. According to the World Health Organization (WHO) and to the Food and Agriculture Organization (FAO) of the United Nations, in Europe it has been estimated that BPA daily intake is around 0.2 μg/kg bw/day for breast-fed babies and around 11 μg/kg bw/day in formula-fed babies for which feeding polycarbonate bottles were used. The estimated daily intake for adults is around 1.5 μg/kg bw/day. Moreover, WHO, Food and Drug Administration (FDA) and European Food Safety Authority (EFSA), based on rodent studies, have also determined the no adverse effect level (NOAEL) for the systemic toxicity of BPA in animals, corresponding, for dietary exposure, to 5 mg/kg bw/day [13, 47]. In consideration of the differences in toxicokinetics among the species, EFSA, on grounds of prudence, applied a default uncertainty factor of 100 to the overall NOAEL and of 1000 to the overall LOAEL, establishing a TDI of 0.05 mg/kg bw (50 μg/kg bw/day) for humans over the life-time [13].

BPA is a lipophilic synthetic organic compound that, following oral administration, is absorbed through the gastrointestinal tract and transported to the liver where it is metabolized acquiring characteristics of hydrophilicity [48, 49]. BPA is metabolized and inactivated through glucuronidation by uridine diphosphate glucuronosyltransferases and sulfation, by phenol-sulfotransferases in hepatocytes microsomes. The conjugated, glucuronidated and sulfated, inactive forms of BPA acquire hydrophilic characteristics and are excreted into the bile and urine, with a half-life corresponding to around 6 hours [47–49]. Nevertheless, the expression of β-glucuronidase enzyme, a member of the glycosidase family of enzymes which cleave the glucuronide group from the metabolite via hydrolysis, in several tissues, like lungs, liver, kidneys and placenta of animals and humans, ensures deconjugation of BPA and therefore the release of its active form into the blood and again its distribution into the body [50]. This mechanism is of a great impact considering that, in mammals, including humans, during pregnancy, conjugated form of BPA cross the placenta, undergo deconjugation, and definitively induce prenatal exposure [51].

BPA active form exerts its primary endocrine disrupting activity mimicking estrogens, but also different hormones, including androgens, such as testosterone (T) and dihydrotestosterone (DHT), and thyroid hormones. Indeed, BPA has a conformational structure that confers the ability to bind both ER alpha (ERα) and beta (ERβ), although, according to in vivo models, the affinity of BPA for ER is 1000-fold to 10,000-fold less than the affinity of 17β-estradiol (E2) [49]. BPA generally induce slow actions through genomic pathways, by directly interacting with nuclear ER and inducing regulation of several genes, generally triggered by direct ER-DNA interactions, or, alternatively, by interacting with gene expression co-regulators; however, BPA can also induce rapid actions through non-genomic pathways, by the activation of kinase signaling cascades via membrane receptors or by the modulation of cell calcium influx variation [52]. Genomic and non-genomic mechanisms can be triggered by BPA low- and high-dose exposure [53].

Approximately 1 to 2.5 ng/mL of active BPA, evaluated by different detection methods (ELISA, HPLC and LC/MS), were absorbed mainly through the primary route (diet) and different sources of exposure (dermal absorption, air inhalation, dust and dental material ingestion), and circulates in human blood [54]. The presence of circulating active BPA has been associated with several human diseases, including metabolic disease (obesity, insulin resistance, diabetes) [47, 55, 56] but has been also associated with female infertility and diseases impairing female fertility [57].

The review will be divided in several sections focused on the following topics: 1) the main epidemiological studies evaluating the association between BPA levels and overall female fertility, in both conditions natural conception and MAR, and 2) the main in vitro, ex vivo and in vivo studies concerning: a) BPA effects on HPO axis; b) BPA effects on female reproductive organs morphology and functions; and 3) the main in vitro, ex vivo, and in vivo studies on BPA role in the development of reproductive disorders, mainly endometriosis and PCOS, including epidemiological studies in humans.

## Bisphenol A and female infertility

Increasing evidences have been suggested that BPA might affect female fertility and could contribute to the pathogenesis of female infertility. In general, infertility affects 25% of couples in developing countries and is defined as the inability to become pregnant after 12 months of regular unprotected sexual intercourse [58]. Couple infertility depends on female factor for around 37%, male factor for around 29% and on combined male and female infertility around 18% of cases, with the remaining 16% due to genetic factors (1%) or due to unknown factors (15%) and therefore indicated as idiopathic infertility (http://old.iss.it/rpma/). The increase in the prevalence of couple, including female infertility, worldwide has been also related to the increased environmental contaminants registered worldwide [59].

The current section describes the present evidence in epidemiological studies concerning the impact of BPA in female infertility, including both natural conception and MAR [60, 61].

### BPA and natural conception

The hypothetical role of BPA in natural conception has been investigated in several observational studies, evaluating the relationship between BPA levels and spontaneous fecundity [62–65].

In the frame of the Italian project PREVIENI, an observational prospective cross-sectional study was performed in 48 women aged 18–40 years affected by infertility aiming to investigate the role of EDCs, including BPA, on reproductive health [62]. The number of subjects with detectable BPA levels (limit of assay detection (LOD): 0.5 ng/ml) was higher in infertile than fertile women [62]. Similar results were reached by an observational prospective case-control study in 153 women aged 18–40 years (43 fertile and 110 infertile women) enrolled in 3 Italian areas representing different living environment scenarios, according to selected territorial, demographic and productive indicators, probably related to different EDCs and BPA exposure patterns: Roma (Lazio, Central Italy), a metropolitan city, with specific metropolitan environment and lifestyle; Ferrara (Emilia-Romagna, Northern Italy), a medium-sized town amid a prosperous area with many farms and small-sized or medium-sized industries; Sora (Lazio, Central Italy), a rural municipality characterized by intensive agricultural activities [64]. Serum BPA levels were detected (LOD: 0.5 ng/ml) in 41.8% of infertile women and 23.3% of fertile women. According to living environment serum BPA levels were detected in 71.4% of infertile women and 23.1% of fertile women in metropolitan area, in 26.3% of infertile women and 27.3% of fertile women in urban area and 4.4% of infertile women and 12.5% of fertile women in rural area, demonstrating that infertile women living of metropolitan area had a trend toward higher percentage of BPA detection compared to infertile women living urban and rural areas and that infertile women had a trend toward higher percentage of BPA detection than fertile women In metropolitan area. The mean of serum BPA levels was 10.6 ng/ml in infertile women and 4.8 ng/ml in fertile women. According to living environment the mean of serum BPA levels were 19.5 ng/ml in infertile women and 7.3 ng/ml in fertile women in metropolitan area, 1.7 ng/ml in infertile women and 2.2 ng/ml in fertile women in urban area, and 6 ng/ml in infertile women and 7.8 ng/ml in fertile women in rural area, demonstrating that infertile women living in metropolitan area had a trend toward higher BPA levels compared to infertile women living in urban and rural areas and that infertile women had a trend toward a higher BPA levels than fertile women living in metropolitan area. Moreover, the higher BPA exposure and levels in infertile women from the metropolitan area might reflect both the greater presence of economic activities employing these chemicals and the characteristic usage patterns of food commodities and consumer products [64]. The Longitudinal Investigation of Fertility and the Environment (LIFE) study, an observational prospective cross-sectional study, evaluated approximately 500 couples (females aged 18–44 and males aged ≥18 years), which discontinued contraception in order to conceive until confirmation of pregnancy or a year of attempts to pregnancy, with the purpose to study the association between BPA exposure and time to pregnancy (TTP). Although the great majority of participants (98%) had urinary BPA levels higher than the LOD (0.05 ng/ml), no association was found between TTP and BPA [63]. However, the results of the study are limited by the fact that BPA levels detected in the study population were much lower than those reported for U.S. biomonitoring data [63]. These findings are consistent with those reported in Maternal Infant Research on Environmental Chemicals (MIREC) study, an observational prospective cross-sectional study on 2001 pregnant women aged 30–40 years recruited in 10 cities across Canada between 2008 and 2011; women were approached during the first trimester of pregnancy at participating hospitals and clinics and were observed for a total of 5 visits up to 10 weeks after delivery [65]. Although detectable urinary BPA levels (LOD: 0.2 ng/ml) were found in 87% of the samples, no association was found between BPA and TTP [65]. It is noteworthy that also in MIREC study BPA levels detected were much lower than those reported in the Canadian Health Measures Surveys of 2009–2011 for women aged 20–39 years, thus probably preventing to find an association between BPA levels and TTP.

Taken together these results suggest that BPA exposure is frequently detected in infertile women and especially in infertile women living in metropolitan area. The level of BPA exposure does not seem to be associated to TTP but the studies investigating this association considering a very low LOD, which was probably one of the main bias preventing to find an association between BPA levels and TTP.

### BPA and MAR

The hypothetical role of BPA in MAR has been investigated in several observational studies reporting a negative relationship between BPA levels and MAR outcomes. Recently it has been highlighted a protective role of soy-based foods against the negative effect of BPA on MAR outcomes. Soy-based foods contain a complex mixture of phytoestrogens, many of which are hormonally active that, like BPA, might have estrogenic activity. The soy-based foods related protective mechanism might lay in the ability to interfere with BPA-induced effects on DNA methylation [66–70].

Bloom et al. investigated the association between serum BPA levels and ovarian response to exogenous gonadotropin stimulation in an prospective cross-sectional carried out in 44 women aged 35–40 years undergoing MAR. An inverse association between serum BPA levels and peak E2 levels were found leading to hypothesize that BPA exposure might influence E2 production during gonadotropin stimulation [66]. Mok-Lin et al. in an observational prospective cohort study carried out in 84 women undergoing MAR found an inverse association of urinary BPA levels with E2 peak and number of oocyte retrieved after gonadotropin stimulation; in particular, for each log unit increase in BPA levels there was an average decrease of 12% in the number of oocytes retrieved and an average of 213 pg/ml decrease in E2 peak after gonadotropin stimulation [67]. Ehrlich et al. in an observational prospective cohort study carried out in 137 women undergoing MAR reported a direct association between urinary BPA levels and implantation failure; in particular, an increased odds of implantation failure was detected in higher quartiles compared with the lowest quartile of urinary BPA levels [68]. Fujimoto et al. in an observational prospective cohort study carried out in 58 infertile female patients aged 36–40 years and 37 male partners aged 38–42 years undergoing a first IVF cycle reported a 9% decrease in the probability to retrieve a mature oocyte for a doubling of female serum BPA levels; further, in the same study a 55% decrease in the probability for fertilization was associated with a doubling in female serum BPA levels [69]. These results were questioned by a recent observational prospective cohort study called the Environment and Reproductive Health (EARTH) Study carried out by Chavarro et al. in 239 women aged 18–45 years who underwent MAR [70]. Participants completed a baseline questionnaire and provided up to 2 urine samples in each treatment cycle before oocyte retrieval. MAR outcomes were abstracted from electronic medical records. The main finding of this study was the association between urinary BPA levels and lower implantation, clinical pregnancy, and live birth rates in women who consumed soy-based foods but not in women who consumed soy-based foods, suggesting a protective role of soy-based from the negative effect of BPA exposure. Although the underlying biological mechanism is still unknown, an action of soy-based foods on BPA-induced effects on DNA methylation has been hypothesized as one of the mechanisms underlying the protective role of soy-based food from the negative effect of BPA [70].

In summary, these findings suggest that BPA could have a negative effect on MAR outcomes, particularly BPA exposure was associated to a decrease in E2 production and number of oocytes retrieved after ovarian stimulation and to an increase in implantation failure. The consumption of soy-based foods might have a protective role against BPA effects on MAR outcome, probably interfering with the BPA-induced effects on DNA methylation.

## Regulation of female reproductive system

Female reproduction in humans and in rodents is closely related to a proper function of HPO axis. Indeed, after sexual maturation, HPO axis coordinates ovarian function and particularly ovarian steroidogenesis and folliculogenesis with the final ovulation and prepares reproductive organs to support a potential pregnancy [3].

Humans and rodents, share the same regulation of reproductive system by the HPO axis, including the regulatory hypothalamic system that releases gonadotropin releasing hormone (GnRH) in rhythmic pulses, the pituitary gland that secretes follicle stimulating hormone (FSH) and luteinizing hormone (LH), and the ovary itself that releases sex hormones, including estrogens, particularly E2, and progesterone (P), which control the function of reproductive system, particularly ovarian and uterine function in the classical cycles [71, 72]. However, species vary significantly in the detailed functioning of ovarian and uterine cycles. Some female primates, including human females, are characterized by a menstrual cycle, in which menstruation occurs in the absence of pregnancy and the females may be sexually receptive at any time during the cycle. Menstrual cycle can be described by the ovarian and uterine cycles. The ovarian cycle refers to a series of changes in ovary during folliculogenesis by which a recruited primordial follicle grows and develops into a specialized Graafian follicle with the potential to be fertilized or to die by atresia. The ovarian cycle consists of the follicular phase, ovulation, and the luteal phase [71, 72]. The uterine cycle refers to a series of changes in the endometrial lining of the uterus and consists in menstruation phase, proliferative phase, and secretory phase. Follicles, located in the cortex of ovary, represent the basic functional unit of reproductive system in females. For humans, follicle development starts during fetal life when primordial follicles are formed. Follicle consists of theca cells that are endocrine cells surrounding the follicle, and of granulosa cells that are somatic cells surrounding the oocyte within the follicle [71]. At the endocrine level the hypothalamus secrets the GnRH to the anterior pituitary in rhythmic pulses by the electrical GnRH neuronal activity. The GnRH pulse generator is the hypothalamic structure that releases GnRH synthesized in specialized neurons [73]. In humans, most GnRH neurons are localized in the mediobasal hypothalamus. GnRH neurons do not express ER and receive E2 signaling from ER-expressing neurons elsewhere within the hypothalamus, such as the kisspeptin (Kiss1) neurons. In humans two major populations of Kiss1 neurons have been identified: one located in the arcuate nucleus (ARC) and another in the preoptic area (POA). Kiss1 neurons send projections to the POA in close proximity to GnRH neurons, stimulating these latter to release GnRH [74]. Secreted pulses of GnRH into the portal blood vessels causes a pulsatile release of FSH and LH, which act on ovary and uterus to control ovarian and uterine cycles [73]. Concentrations of LH and FSH vary throughout the menstrual cycle. In the early follicular and luteal phases FSH is predominant over LH, whereas LH is dominant over FSH in the late follicular phase [73]. During follicular phase of ovarian cycle and proliferative phase of uterine cycle, the activated primordial follicles with a single layer of granulosa cells surrounding the primordial oocytes develop into primary, secondary, and eventually antral follicles under FSH stimulation. A few of antral follicles reach the preovulatory stage, whereas most antral follicles undergo atretic degeneration. In antral follicle LH stimulates the conversion of cholesterol into androgens in the theca cells, thereby increasing endogenous intra-ovarian androgen production, in particular T. At the same time, FSH stimulates the expression and activity of aromatase in granulosa cells, inducing the conversion of androgens into estrogens, mainly E2. The E2 released by antral follicle reaches the maximum circulating level in the preovulatory stage to further regulate follicular maturation, increasing growth and differentiation of granulosa cells, and to regulate the HPO with a negative feedback mechanism [75, 76]. Increased E2 production induces the thickening of endometrium, the uterine inner epithelial layer, along with its mucous membrane, and activates Kiss-1 neurons with a consequent increased of GnRH pulse frequency and amplitude. Fast GnRH pulse frequencies induce LH synthesis leading to the LH surge [73]. The spike in LH causes ovulation during which the dominant preovulatory follicle ovulates to release the mature oocyte for fertilization. Following ovulation, in the luteal phase, the remaining theca and granulosa cells undergo transformation to become the corpus luteum that produces P as well as E2 [71]. Luteinization of the granulosa cells increases P production, acting to stabilize the endometrium at the optimal thickness to support implantation, to become receptive to the fertilized egg and to prepare the endometrium for the potential the egg implantation, and to thrive for the duration of the pregnancy and slowing GnRH pulse frequency that in turn decreases LH production and increases FSH production to stimulate the next round of ovulation. If fertilization does not occur, the corpus luteum will start to break down resulting in a drop in E2 and P levels, which induce menstrual discharges, due to the shedding and collapse of the endometrium. Under the FSH-dependent estrogens stimuli uterine lining thickens and the cycle begins again [71, 73].

Non-primate females, including rodent females, instead, are characterized by an estrus cycle, in which the endometrium is reabsorbed if conception does not occur during the cycle and in which there are recurring periods when the females are fertile and sexually receptive (estrus) interrupted by periods in which the females are not fertile and not sexually receptive (anestrus) [77]. Estrous cycles start after puberty in sexually mature females and typically continue until death; rodents undergo estrus cycles throughout the whole year [77]. In rodents, follicle development starts during neonatal life when primordial follicles are formed. In rodents, GnRH cell bodies reside in the POA and rostral hypothalamus and Kiss1 neurons in the rostral periventricular area of the third ventricle (RP3V). RP3V in rodents consists of Kiss1 cells clustered in the anteroventral periventricular nucleus (AVPV) that extend caudally into the adjacent periventricular preoptic zone (PeN) [74]. Kiss1 neurons send projections to the RP3V in close proximity to GnRH neurons, stimulating these latter to release GnRH. Many evidences indicate that Kiss1 neurons in RP3V-AVPV regulate GnRH/LH-surge generation, while ARC Kiss1 neuronal population regulates GnRH pulse generation [74, 78]. Hypothalamic regulation of rodent reproductive cycle is the same described for humans. Differently from humans, in female of rodents, reproductive processes, are characterized by cyclic morphological changes in female reproductive system and cyclic sexual receptivity [77]. The recurrent period of receptivity, or “heat” is called estrus. The entire estrus cycle, that occurs over 4–5 days, is formed by four stages: 1) diestrus, during which in ovary small follicles are present with large corpora lutea from the previous ovulation, the uterus is atrophic and with low motility. During this stage E2 levels start to increase whereas FSH and LH levels are low; 2) proestrus, during which ovarian follicles grow rapidly and the uterus is hypertrophic and with a pronounced contractility. This stage corresponds to pre-ovulatory day characterized by increased E2 and P levels and the occurrence of the ovulation after LH and FSH surges; 3) estrus, during which ovulation of more than 15 eggs occur and the uterus reach the maximum development of endometrium. E2 levels remains elevated during the morning and fall down in the afternoon; and 4) metestrus, during which many corpora lutea secrete P only for a short time and the uterus decrease in size and in vascularity with the degeneration and replacement of endometrium. E2, LH and FSH levels are low [77].

## Bisphenol A and HPO axis

The HPO axis plays a critical role in the development and regulation of reproductive system. Fluctuations in this axis cause changes in the hormones produced by each gland and triggering various local and systemic effects. GnRH is secreted from the hypothalamus by GnRH-expressing neurons and activates the anterior portion of the pituitary gland to produce LH and FSH. Both gonadotropins stimulate the ovary to produce E2 and T. The following section describes the available data on the putative effects, and underlying mechanisms, of BPA on HPO axis, obtained in experimental in vitro, ex vivo and in vivo studies. The studies will be described dividing the target of HPO axis, such as hypothalamus, pituitary and ovary. The studies reporting the effects of BPA on HPO axis, with impact on reproductive function, particularly ovarian steroidogenesis, and, therefore, on female fertility are summarized in Tables 1, 2 and 3.

**Table 1:** Bisphenol A and hypothalamus-pituitary-ovary (HPO) axis: hypothalamus

	Source	Strain	Age	Exposure route	Time of exposure	Doses	Time of observation	Outcome	Outcome observed in	Reference n°	
Experimental studies in vitro and ex-vivo	Rat	Sprague-Dawley	Female Pups	Subcutaneous Injection	From PND1 to PND10	6.2–2.5 mg/kg bw/day and 62.5–25.0 mg/kg bw/day	PND13	Increased GnRH pulsatility in hypothalamic explants	Adult female	Fernandez 2009	[79]
	Rat	Sprague-Dawley	Female pups	Subcutaneous Injection	From PND1 to PND10	5, 50 and 500 μg/kg bw/day	PND13	Adult female rats postnatally exposed to BPA presented alterations in GnRH secretion, as demonstrated by a decrease in the IPI	Adult female and female pups	Fernandez 2010	[80]
Experimental studies in vivo	Mouse	CD-1	Adult pregnant mice	Oral gavage	From GD1 to PND20	12, 25 and 50 mg/kg bw/ day	PND50	Increased Kiss1 and GnRH mRNA expression at 25–50 mg/Kg bw/day during proestrus phase	Female offspring (F1) in adulthood	Xi 2011	[29]
	Mouse	CD-1	Female pups	Oral gavage	From PND20 to PND49	25 and 50 mg/kg bw/ day	PND50	No effect on Kiss1 and GnRH mRNA expression during proestrus phase	Female offspring (F1) in adulthood	Xi 2011	[29]
	Mouse	ICR	Adult female mice	Oral administration	Proestrus after 4th/5th estrus cycle	20 μg/kg bw/day	6 h after BPAadministration	Increased GnRH mRNA expression in POA as well as Kiss1 mRNA in AVPV, but not Kiss1 in ARC during proestrus	The same adult female mice (12- week-old)	Wang 2014	[78]
	Mouse	ICR	Adult female mice	Injection into the right lateral ventricle	Proestrus after 4th/5th estrus cycle	0.02, 0.2, 2.0, 20.0, and 200.0 nM/3 ml	6 h after BPA administration	Increased GnRH mRNA expression in POA. Increased Kiss1 mRNA in AVPV and decreased Kiss1 mRNA in ARC during proestrus	The same adult female mice (12- week-old)	Wang 2014	[78]
	Mouse	C57BL/6J	Adult second-pregnancy dams	Oral gavage	Pregnancy (gestational day 15) and lactation (PND21) period	0.05 and 5 mg/kg bw/ day	During diestrus 5 weeks after BPA administration	Increased Kisspeptin protein expression in RP3V but no change in GnRH protein in the POA during diestrus	Female offspring in adulthood (from 8 weeks of age)	Naule 2014	[81]
	Rat	Wistar-derived strain	Female Pups (F1)	Subcutaneous Injection	From PND1 to PND7	0.05 and 20 mg/kg bw/ day	During estrus from PND85 to PND100	Increased GnRH mRNA levels at 0.05 mg/kg bw/day and decreased at 20 mg/kg bw/day during estrus	Female offspring in adulthood (PND100)	Monje 2010	[82]
	Rat	Long Evans	Female Pups (F1)	Subcutaneous Injection	From PND1 to PND3	50 μg/kg bw/ day and 50 mg/kg bw/ day	7 weeks after PND148	No impairement of GnRH neurons activation	Female pups (F1)	Adewale 2009	[83]
	Monkey	*Macaca mulatta*	Pubertal female	Infusion in stalk-median eminence	During follicular phase	0.1, 1 and 10 nM	Each 20’ for 240’ after BPA exposure	Decreased GnRH and Kisspeptin secretion and pulse after 10nM of BPA at puberty	Pubertal female	Kurian 2015	[84]
	Sheep	n.r.	Adult pregnant sheep	Subcutaneous Injections	From GD 30 to GD90	5 mg/kg/day	During estrus in sheep at 21 months of age	Decreased GnRH mRNA expression in hypothalamus. Increased of ERα mRNA expression and reduced ERβ mRNA expression in POA.	Female Offspring in adulthood	Mahoney 2010	[85]

**Table 2:** Bisphenol A and hypothalamus-pituitary-ovary (HPO) axis: pituitary

	Source	Strain	Age	Exposure route	Time of exposure	Doses	Time of observation	Outcome	Outcome observed in	Reference n°	
Experimental studies in vitro and ex-vivo	Rat	Sprague-Dawley	Female pups	Subcutaneous Injection	From PND1 to PND10	6.2–2.5 mg/kg bw/day and 62.5–25.0 mg/kg bw/day	PND13	Decreased basal and GnRH-stimulated LH levels in medium of adult anterior pituitary cell cul tures. Adult anterior pituitary cell cultures treated with BPA 6.2–2.5 mg/kg bw/day showed rapid and transient activation of ERK1/2 com pared to control	Female rats in adulthood in estrus phase	Fernandez 2009	[79]
Experimental studies in vivo	Mouse	CD-1	Adult pregnant mice	Oral gavage	Cohort A: from GD1 to PND20	12, 25 and 50 mg/kg bw/day	PND50	Increased FSH mRNA expression. No significant change in serum LH and FSH Levels at proestrus phase	Female offspring (F1) in adulthood	Xi 2011	[29]
	Mouse	CD-1	Female pups (F1)	Oral gavage	Cohort B: from PND20 to PND49	25 and 50 mg/kg bw/day	PND50	No significant change in LH and FSH mRNA expression at proestrus phase	Female offspring (F1) in adulthood	Xi 2011	[29]
	Rat	Sprague-Dawley	8-week-old	Oral gavage	From GD1 to PND20	0.001 or 0.1 mg/kg bw/ day	After 90 days of exposure	Increased serum LH levels and LH protein content after BPA exposure of 0.001 and 0.1 mg/kg bw/ day. No significant change in serum FSH levels and FSH protein expression during estrus	The same adult female rats (8-week- old)	Lee 2013	[30]
	Rat	Wistar	Adult pregnancy rats	Oral administration (drinking water)	From GD1 to PND21	3 μg/kg bw/Day	PND30	Increased serum LH levels. No significant change in serum FSH levels	Female offspring in prepubertal phase	Gamez 2015	[31]
	Rat	Sprague-Dawley	Female pups	Subcutaneous Injection	From PND1 to PND10	6.2–2.5 mg/kg bw/day and 62.5–25.0 mg/kg bw/day	PND13	Decreased basal and GnRH-stimulated serum LH levels at BPA 62.5–25.0 mg/kg bw/day.	Female pups	Fernandez 2009	[79]
	Rat	Sprague-Dawley	Female pups	Subcutaneous Injection	From PND1 to PND10	6.2–2.5 mg/kg bw/day and 62.5–25.0 mg/kg bw/day	During estrus	Decreased GnRH-stimulated (15′) serum LH levels at BPA 62.5–25.0 mg/kg bw/day during estrus	Female rats in adulthood	Fernandez 2009	[79]
	Mouse	ICR	Adult female mice	Oral Administration	Proestrus after 4th/5th estrus	20 μg/kg bw/ day	During diestrus, proestrus and estrus	Increased serum LH and FSH levels during proestrus	The same adult female mice	Wang 2014	[78]
	Mouse	ICR	Adult female mice	Injection into the right lateral ventricle	Proestrus after 4th/5th estrus	0.02, 0.2, 2,20 and 200nM/3μl	During diestrus, proestrus and estrus	Increased serum LH levels during proestrus	The same adult female mice	Wang 2014	[78]
	Rat	Sprague-Dawley	Adult Female rats	Oral Administration	After two classic estrous cycles	50 mg/kg bw/day	6 consecutive weeks	Increased serum LH and FSH levels	The same adult female rats	Zhou 2014	[86]
	Mice	C57BL/6J	Adult female mice	Oral administration	12–15 days (first 3 reproductive cycle)	50 μg/kg bw/ Day	51–54 days	No significant change in serum LH and FSH Levels	The same adult female mice	Moore- Ambriz 2015	[87]
	Rat	Sprague- Dawley	Adult Female rats	Oral gavage	42 days	10 mg/kg bw/day	After the last treatment day, during diestrus	Decreased serum LH and FSH levels during diestrus	The same adult female rats	Zaid 2018	[88]
	Rat	Wistar	Adult female	Subcutaneous Injection	From PND 90 to PND105	25 ng/kg/d and 5 mg/kg/ day	24 h after last treatment, during diestrus	No significant change in serum LH and FSH levels. Delay in LH surge during diestrus	The same adult female rats	Lòpez-Rodríguez 2019	[89]
	Mouse	C57BL/6J	Adult second- pregnancy mice	Oral gavage	Pregnancy From GD15 to lactation (PND21) period	0.05 and 5 mg/kg bw/ day	During diestrus 5 weeks after BPA administration	No change in serum LH levels during diestrus	Female offspring in adulthood	Naule 2014	[81]
	Rat	Wistar-derived strain	Female Pups (F1)	Subcutaneous Injection	From PND1 to PND7	0.05 and 20 mg/kg bw/ day	During estrus from PND85 to PND100	Precluded the production of an E2-induced LH surge at 20 mg/kg bw/day during estrus	Female pups in adulthood from PND85 to 100	Monje 2010	[82]

**Table 3:** Bisphenol A and hypothalamus-pituitary-ovary (HPO) axis: ovary

	Source	Strain	Age	Exposure route	Time of exposure	Doses	Time of observation	Outcome	Outcome observed in	Reference n°	
Experimental studies in vitro and ex-vivo	Rats	Ovarian theca- interstitial (T-I) obtained from Sprague– Dawley rats	28–30 days old	In vitro administration	72 h	BPA low concentration 10–7 M and high concentration from 10 to 4 M to 10–6M	After 72 h	Increase cholesterol side-chain cleavage enzyme (P450scc) and 17 alpha-hydroxylase/17,20 lyase (P450c17) mRNA at all BPA concentrations tested. Increased StAR mRNA expression and T secretion at BPA 10–5 and 10–4M	Ovarian theca- interstitial (T-I) obtained from Sprague– Dawley rats	Zhou 2008	[28]
	Rats	Granulosa cells obtained from Sprague– Dawley rats	28–30 days old	In vitro administration	72 h	from 10 to 4 M to 10–7M	After 72 h	Increased StAR mRNA and P levels in cell media from BPA 10–7 to 10–5 M. Decreased P levels in cell media at BPA 10–4 M. E2 levels dose-dependently decreased while aromatase mRNA increased after BPA at 10–7 to 10–4M	Granulosa cells obtained from Sprague– Dawley rats	Zhou 2008	[28]
	Mice	FVB	Adult female	Ex vivo administration in antral follicles	PND32	4.4, 44, and 440 μM	120 h	Decreased levels of P and E2 in cell media at 440 μM of BPA. Decreased StAR and 3ß-HSD mRNA expression in antral follicules after BPA exposure of 440 μM	Antral follicules of adult female mice	Peretz 2011	[32]
	Human	Luteinized granulosa cells	Fertile and infertile patients < 38 years old	In vitro Administration	48 h	0.2, 0.02,2.0, 20 μg/ml	After 48 h	P and E2 reduced secretion in cell media. P450scc, 3ß-HSD and aromatase mRNA expression reduced at BPA higher concentrations.	Luteinized granulosa cells	Mansur 2016	[33]
	Mice CD-1	Antral follicles	PND32-PND35	In vitro administration	24h-96h	1.0. 10, 100μg/ml	24h-96h	Lack of cholesterol conversion to Pregnenologne and consequently decreased of CYP11a1 and StAR expression. Decrease of androsteneidione, T, and E2 levels	Antral follicles	Peretz 2013	[34]
Experimental studies in vivo	Mouse	CD-1	Adult pregnant mice	Oral gavage	Cohort A: From GD1 to PND20	12, 25 and 50 mg/kg bw/day	PND50	Increased serum E2 levels associated with mRNA and protein P450scc and aromatase expressions up- regulation after BPA exposure to 12, 25 and 50 mg/kg bw/day in proestrus phase	Female offspring (F1) in adulthood	Xi 2011	[29]
	Mouse	CD-1	Female pups (F1)	Oral gavage	Cohort B: From PND20 to PND49	25 and 50 mg/kg bw/day	PND50	Increased serum E2 levels wherease no change in CYP mRNA expression at proestrus phase	Female offspring (F1) in adulthood	Xi 2011	[29]
	Rat	Sprague- Dawley	8-week-old	Oral gavage	90 days	0.001 or 0.1 mg/kg bw/day	After 90 days of exposure during estrus	Decreased serum E2 levels. Decreased aromatase protein expression in granulosa cells in particular after BPA exposure of 0.001 mg/kg bw/day. Downregulation of StAR protein expression after BPA exposure of 0.001 or 0.1 mg/kg bw/day	The same adult female rats (8-week- old)	Lee 2013	[30]
	Rat	Wistar	Adult pregnant rats	Oral administration (drinking water)	From GD1 to PND21	3 μg/kg bw/day	PND30	Increased serum E2 levels	Female offspring (F1) in prepubertal phase	Gamez 2015	[31]
	Mice	ICR	Adult Female mice	Oral administration	6h	20μg/kg bw/ day	Diestrus, proestrus and estrus	Increased serum E2 levels during proestrus	The same adult mice	Wang 2014	[78]
	Mice	ICR	Adult female mice	Injection into the right lateral ventricle	6h	0.02, 0.2, 2,20 and 200 nM/3μl	Diestrus, proestrus and estrus	Increased serum E2 levels during proestrus	The same adult mice	Wang 2014	[78]
	Rat	Sprague-Dawley	Female pups	Subcutaneous Injection	From PND1 to PND10	6.2–2.5 mg/kg bw/day and 62.5–25.0 mg/ kg bw/day	PND13	Increased levels of serum E2 and T, and decreased P serum levels	The same rats in adulthood	Fernandez 2010	[80]
	Rats	Sprague-Dawley	Adult female	Oral administration	After two classic estrous cycles	50 mg/kg bw/ day	6 consecutive weeks	Increased mRNA and protein expression of FSHR	The same adult female rats	Zhou 2014	[86]
	Mice	C57BL/6 J	39 days old	Oral administration	12–15 days (first 3 reproductive cycle)	50 μg/kg bw/ day	At third proestrus	No significant change in serum E2 levels	The same adult female mice (39-days-old)	Moore-Ambriz 2015	[87]
	Ewes	Suffolk	N.R.	Injection	From GD30 to GD90	0.5 mg/k	From GD30 to GD90	Prenatal BPA increased Cyp19 and 5α-reductase expression in day 65, but not day 90, ovaries. Fetal ovarian microRNA expression was altered by prenatal BPA with 45 down-regulated at day 65 and 11 down-regulated at day 90 of gestation. These included microRNAs targeting Sry- related high-mobility-group box (SOX) family genes, kit ligand, and insulin-related genes	Female offspring at fetal day 65 and at fetal day 90	Veiga- Lopez 2013	[90]
	Rat	Sprague- Dawley	28 days-old	Oral gavage	42 days	10 mg/kg bw/ day	After the last treatment day, during diestrus	Slight but not significant increase of E2 serum levels and reduction of P serum levels	The same adult female rats (70-days-old)	Zaid 2018	[88]
	Mouse	C57BL/6 J	Adult second- pregnancy mice	Oral gavage	Pregnancy GD15) and lactation (PND21) period	0.05 and 5 mg/kg bw/day	During diestrus 5 weeks after BPA administration	Increased serum E2 levels in diestrus	Female offspring in adulthood	Naule 2014	[81]

*N.R* not reported

### Experimental studies in vitro and ex vivo

Few experimental in vitro and ex vivo studies have investigated the effects of BPA exposure on HPO axis regulation [28, 32, 33, 79, 80].

#### Hypothalamus

Ex vivo studies conducted on cell primary cultures of POA-anterior hypothalamus-medial basal hypothalamus showed that hypothalamic cells, explanted at PND13 or at adulthood by rat female pups postnatally exposed, from PND1 to PND10, to subcutaneous administrations of low and high BPA doses, exhibited a higher GnRH pulse frequency and decreased GnRH inter-pulse intervals [79, 80].

#### Pituitary

An ex vivo study conducted on primary cultures of pituitary gland explanted at PND13 by rat female pups postnatally exposed, from PND1 to PND10, to subcutaneous administrations of low and high BPA doses showed a reduced basal and GnRH-stimulated LH release exclusively in cells exposed at high BPA doses [79]. The intracellular signaling involved in the regulation of LH release by BPA seems to implicate ERK pathway. Indeed, GnRH-stimulated pituitary cells, explanted in adulthood by rat female pups postnatally not exposed, exhibited a rapid and sustained ERK1/2 phosphorylation; conversely, GnRH-stimulated pituitary cells, explanted in adulthood by rat female pups postnatally exposed, from PND1 to PND10, to low and high BPA doses, showed a rapid but transient activation of ERK1/2 [79].

#### Ovary

In primary cultures of rat theca-interstitial cells, treatment with low and high BPA doses increased steroid 17 alpha-hydroxylase/17,20 lyase (cytochrome P450c17), cholesterol side-chain cleavage enzyme (P450scc) and steroidogenic acute regulatory (StAR) messenger expression, as well as T secretion. In particular, StAR messenger expression increased only at high BPA doses and T secretion at very high tested BPA dose, suggesting that BPA may induce hyperandrogenism, especially at higher BPA doses [28]. In primary cultures of rat granulosa cells, treatment with low and high BPA doses dose-dependently decreased aromatase messenger expression together with E2 secretion [28], but increased StAR messenger expression together with P secretion that, surprisingly, markedly decreased at very high BPA doses with a concomitant decrease of P450scc messenger expression [28]. Conversely, in primary cultures of human luteinized-granulosa cells explanted by women undergoing to in vitro fertilization, high BPA doses reduced E2 and P secretion and messenger expression of P450scc, 3-beta-hydroxysteroid dehydrogenase (3ß-HSD) and aromatase [33]. Finally, in follicle cultures, obtained by ex vivo explant of antral follicles mechanically isolated from mice ovary, short and long-term treatment with high BPA doses induced a decrease of E2 and P secretion with a concomitant decrease of StAR, 3ß-HSD and Cyp11a1 messenger expression [32, 34].

### Experimental studies in vivo

Several experimental in vivo studies have investigated the effects of BPA exposure on HPO axis regulation [29–31, 78–90].

#### Hypothalamus

The effect of BPA on the hypothalamic hormone secretion, messenger expression and neuronal activity has been investigated in female pups and adult animals during different phases of estrus cycle. Oral administration of low BPA doses markedly increased GnRH messenger expression in POA as well as AVPV-Kiss1, but not ARC-Kiss1, messenger expression, while intracerebroventricular administration increased also ARC-Kiss1 messenger expression, during proestrus, but not during diestrus and estrus, in female adult mice [78], providing in vivo evidence that exposure of female mice to low BPA doses in adulthood can disrupt the HPO axis. Harmful effects of BPA exposure have been also demonstrated in adulthood when female mice were continuously exposed to low BPA from the prenatal period through postnatal period till adulthood. Indeed, mouse female offspring, whose dams were exposed during pregnancy from GD1 to delivery, perinatally and postnatally exposed, from day PND1 to PND49, by gavage of low BPA doses, showed a dose-dependent increase in Kiss1 and GnRH messenger expression in the hypothalamus in adulthood [29], or an increased Kiss1 protein expression in the RP3V, but not GnRH protein expression in the POA, during diestrus in adulthood [81]. Deleterious effects of BPA on HPO axis have been also demonstrated when the exposure is restricted to the postnatal period, although the results referring to this window of BPA exposure are conflicting. Indeed, in rat female pups postnatally exposed, from PND1 to PND7, to low BPA doses by subcutaneous administration induced an increase of hypothalamic GnRH messenger expression during estrus in adulthood [82]. However, in mouse and rat female pups postnatally exposed to low BPA doses by gavage, from PND20 to PND49, or by subcutaneous administration, from PND1 to PND3 [83], no alteration of hypothalamic Kiss1 and GnRH messenger expression was found during estrus in adulthood [29]; moreover, in the same setting, no impairment of GnRH neurons activation, essential to generate pulses was reported after the P injection [83]. Direct effects of BPA exposure have been also evaluated on female primate hypothalamic hormone release; indeed, low BPA doses infusion in stalk-median eminence suppressed GnRH and Kiss1 release in hypothalamus of female pubertal monkeys [84]. In a different animal model, namely in sheep female pups, perinatally exposed to subcutaneous administration of low BPA doses, decreased hypothalamic GnRH messenger expression and increased ER messenger expression was reported during the late follicular phase following estrus synchronization in adulthood [85].

#### Pituitary

The effect of BPA on the pituitary hormone secretion and messenger expression has been investigated in female pups and adult animals during different phases of estrus cycle. Oral administration of low BPA doses in female adult mice and rats increased serum FSH and LH levels during proestrus [78, 86], while increased only serum LH levels during proestrus when exposed to subcutaneous administration of low BPA doses [78]. Moreover, female adult rats exposed to gavage of low BPA doses showed increased serum LH levels and LH protein content in the pituitary gland, with no change in FSH levels and FSH protein content, during estrus [30]. In rat female offspring perinatally and postnatally exposed, from GD 1 to PND21, to oral administration of low BPA doses increased serum LH levels were observed during prepubertal period [31], while higher BPA doses, although still in the range of low doses, precluded the production of an E2-induced LH surge, during estrus, in rat female pups postnatally exposed, from PND1 to PND7, by subcutaneous administration [82]. Furthermore, in rat female pups postnatally exposed, from PND1 to PND10, to subcutaneous administration of high BPA doses decreased GnRH-induced serum LH release at PND13 and in adulthood during estrus; this effect was not found at low BPA doses [79]. Conversely, several studies reported that prenatal and postnatal exposure to low BPA doses reduced serum LH and FSH levels [88] or that exposure to low BPA doses with different administration routes did not affect either circulating LH [29, 81, 87, 89] or FSH [29–31, 79, 87, 89] levels in female adult mice and rats observed in different phases of estrus cycle. Finally, an up-regulation of FSH, but not LH, messenger expression has been observed in mouse female offspring perinatally exposed by gavage administration to low BPA doses [29].

#### Ovary

The effect of BPA on the sex hormone secretion and messenger expression has been investigated in female pups and adult animals during different phases of estrus cycle. Indeed, in rat and mouse female offspring perinatally, prenatally and postnatally exposed to oral, gavage and subcutaneous administration of low and high BPA doses increased circulating E2 levels have been recorded both at PND13 and PND50 in prepubertal or at adulthood, during proestrus and diestrus phases [29, 31, 80, 81]. Increased E2 circulating levels have been also reported in adult mice after acute low BPA administration [78]. Conversely, in further studies conducted on female adult mice and rats treated with low BPA doses by oral and gavage administration, no changes in circulating E2 levels have been reported during proestrus and diestrus [87, 88]. Moreover, in female adult rats exposed in adulthood to low BPA doses by gavage administration decreased circulating E2 levels have been found during estrus [30]; the decrease in circulating E2 levels in female adult rats exposed to low BPA doses have been associated to a down-regulation of StAR and aromatase protein synthesis, as confirmed by immunohistochemical studies on ovary [30]. In female adult rats postnatally exposed to subcutaneous administration of low and high BPA doses an increase of circulating T levels and a decrease of circulating P levels has been detected in adulthood [80]. BPA exposure also affects the expression of several steroidogenic enzymes. Indeed, concomitant perinatal and postnatal exposure by gavage administration to low BPA doses of mouse female pups increased P450scc and aromatase messenger and protein expression [29]. Increased aromatase and 5α-reductase messenger expression has been also observed in fetal ovaries at 65 days of gestation of pregnant Suffolk ewes after treatment with low BPA doses [90]. In addition to the regulation of sex hormone synthesis, exposure to low BPA doses increased messenger and protein expression of FSH receptor (FSHR) in ovarian tissue of female adult rats [86].

### Observational studies in humans

Although BPA has been found to impair HPO axis in animal studies, no study in humans has been investigated the effect of BPA on HPO axis.

Taken together, these findings demonstrate that exposure to low and high BPA doses has harmful effects on the HPO axis causing hormone dysregulation during the different phases of the estrus cycle. However, it is noteworthy that the available data reports discordant results. The different outcome might likely depend on animal model, BPA doses, administration route and window of exposure. Indeed, although BPA deleterious effects have been reported to be more critical with perinatal exposure, during embryonic development, compared to postnatal exposure, BPA exposure during postnatal period or in adulthood can also negatively interfere with HPO axis. Moreover, a different route of administration can differently regulate HPO axis in the same phase of estrus cycle of the same animal model. According to results of ex vivo and in vivo studies on rodent models, postnatal and, particularly, prenatal BPA exposure induces a precocious maturation of the HPO axis by generally increasing GnRH and Kiss1 messenger and protein expression and GnRH pulsatility in the hypothalamus during different estrus cycle phases, whereas according to the results of in vivo studies in monkeys and sheeps, BPA perinatal exposure or BOA exposure during pubertal phase negatively regulate GnRH messenger expression and release, demonstrating that BPA effects on hypothalamus can vary depending on window of exposure and animal models. Similarly, variable results have been found in the analysis of pituitary secretion; since according to the results of ex vivo and in vivo studies in rodent models BPA exposure can cause a different modulation of gonadotropins, that are generally increased, particularly LH, during prepubertal and in adulthood, proestrus and estrus phases, but may also be unchanged or decreased, especially after GnRH stimulation. It is noteworthy that some studies reported different results in the same model and estrus phase but with different route of exposure, suggesting that the administration route of BPA has a key role in the manifestation of the BPA deleterious effects in HPO axis. Finally, according with the results of ex vivo human and rodent models and in vivo rodent models BPA perinatal and postanatal exposure can cause a different modulation of sex hormone secretion, with impairment of ovarian expression of steroidogenic enzymes involved in sex hormone production and consequent increase of T secretion and decrease of P secretion, in diestrus and estrus phases, and generally an increase of E2 secretion in estrus phase, although even unchanged or decreased E2 levels have been reported.

## Bisphenol A and reproductive system morphology and functions

The female reproductive system includes the paired ovaries, the oviducts, the uterus, and the vagina. These organs are finely tuned and coordinated to perform the primary functions reproductive system, including folliculogenesis, fertilization, and support of the development of embryo, and to allow the birth of fetus. Morphological and functional changes of reproductive system, particularly the alterations of ovary and uterus, can impair female fertility. In human and rodents, the ovaries are composed by a central highly vascular medulla surrounded by a cortex in which ovarian follicles can be found at different stages of development. The morphological alterations of ovaries can take place during ovarian development or after puberty in fertile-aged females, such as in specific reproductive diseases, like polycystic ovary syndrome (PCOS). In humans and rodents, ovarian development is a dynamic process consisting in the growth of the ovary and establishment of the finite pool of primordial follicles, occurring predominantly during the embryonic period [91]. In particular, in humans, ovarian development is sustained for years while in rodents it can occurs in mere weeks, beginning on E5 and finishes on PND2 [92]. During embryonic development, in humans and rodents, primordial germ cells, precursors of oocytes, undergo extensive mitotic proliferation, maintain pluripotency and form clusters termed germ cell nests, surrounded by a single layer of granulosa cells, and then enter meiosis to form oocytes. Approximately 70% of the oocytes in the germ cell nests die, a process that leads to germ cell nest breakdown and allows the formation of individual primordial follicles. Primordial follicles represent a finite pool of resting oocytes, namely the entire ovarian reserve, available during their entire reproductive lifespan [91, 92]. In humans and rodents, the functional alterations of ovary, which can be cause or consequence of alterations of ovarian morphology, include mainly the impairment of folliculogenesis, beyond the impairment of steroidogenesis and sex hormones production. Humans have a pear-shaped uterus with a single triangular-shaped cavity. Rodents have a bicornuate uterus consisting of two lateral horns (cornua) that join distally into a single body (corpus). Hystological examination revealed that in both human and rodents the uterus is composed of three tissue layers: 1) the endometrium, the inner and functional layer responding to reproductive hormones; 2) the myometrium, the intermediate layer composed of smooth muscle cells; and 3) the perimetrium, the thin outer layer composed of epithelial cells [93]. In humans and rodents, the functional alterations of uterus, which can be consequence of morphological uterine alterations, include mainly the uterine receptivity [94].

The following section describes the available data on the putative effects, and underlying mechanisms, of BPA on the morphology and function of female reproductive organs, in particular on ovary and ovarian folliculogenesis and on uterus and embryo implantation, obtained in experimental in vitro, ex vivo and in vivo studies. The studies reporting the effects of BPA on morphology and functions of reproductive system organs and, therefore, on female fertility are summarized in Table 4.

**Table 4:** Bisphenol A and reproductive system morphology and functions

	Source	Strain	Age	Exposure route	Time of exposure	Doses	Time of observation	Outcome	Outcome observed in	Reference n°	
Experimental studies in vitro and ex vivo	Mouse	Sensitivity to FVB	32-day-old	In vitro administration	24–120 h	4.4, 44, and 440 μM	24–120 h	BPA inhibits follicle growth and decreases hormone production in mouse ovarian antral follicles. Pregnenolone protected follicles from BPA-induced inhibition of steroidogenesis.		Peretz 2011	[32]
	Mouse	Kunming	N.R.	Gavage	From GD0.5 to 3.5	200, 400, 600, and 800 mg/kg/day	From GD0.5 to 3.5	Increase of eNOS protein expression. Remarkably reduced the number of implantation sites of pregnant mice.		Pan 2015	[37]
	Mouse	CD-1	N.R.	In vitro administration	From PDN 0 to PDN 10	0,1, 1 and 10 μM	From PDN 0 to PDN 10	Enhanced primordial follicle recruitment by decreasing Ki-67 and caspase-3 expression and by activating PI3K/AKT pathway.		Zhao 2014	[95]
	Mouse	CD-1	PND 0	In vitro administration	For 1–8 days	0.1, 1, 5, and 10 μg/ mL	For 1–8 days	Inhibition of germ cell nest breakdown increasing expressione of Bcl2 and decreasing of FAS and caspase 8		Zhou 2015	[96]
	Mouse	C57/Bl6JxCBA/ Ca	12–14 day-old	In vitro administration	At the start of follicle culture and each replenishment for 13 days	3 nM and 300 nM	At the start of follicle culture and each replenishment for 13 days	Accelerated follicle development with an increase in antral follicle growth.		Trapphoff 2013	[35]
	Mouse	Sensitivity to FVB	2- to 35-day-old	In vitro administration	24–96 h	1, 10, and 100 μg	24–96 h	Estradiol does not protect follicles from BPA- induced growth inhibition and does not protect follicles from BPA-induced atre sia. BPA up-regulates Cdk4, Ccne1, and Trp53 expression, whereas it down- regulates Ccnd2 expression. BPA also up- regulates Bax and Bcl2 expression while in ducing atresia in antral follicles.		Peretz 2012	[36]
	Mouse	CD-1	PND 32– 35	In vitro administration	24–96 h	1.0, 10, and 100 μg/mL	24–96 h	Lack of cholesterol conversion to pregnenolone and consequently decrease of Cyp11a1 and StAR expression. Decrease of androstenedione, testosterone, and estradiol levels.		Peretz 2013	
	Mouse	C57BL/6	50–54 days old	In vitro administration	24–96 h	0.004, 0.04, 0.44, 4.38, 43.8, 110, 219 and 438 μM	24–96 h	BPA inhibited follicle growth and decreased estradiol levels		Ziv-Gal 2013	[97]
	Mouse	C57BL6	2–3 months old	Subcutaneous Injiecton	From GD0.5 to GD3.5.	0, 0.025, 0.5, 10, 40, and 100 mg/kg/day	From GD0.5 to GD3.5.	Females treated with 100 mg/kg/day BPA, did not show implantation sites on day 4.5. In 40 mg/kg/day BPA treated females.	Female offsprings in adulthood	Xiao 2011	[38]
	Mouse	CF-1	3–5 months	Subcutaneous Injections	From GD1 to GD4	100, 200, and 300 mg/kg	From GD1 to GD4	Disruption of intrauterine implantation and alteration in uterine morphology. Expansion in uterine luminal area and an increase in luminal epithelial cell height. ER alpha and PR expression was modulated as a non-monotonic function of BPA dose, with some evidence of a rise with the lowest dose and declines with increasing dose.	Adult female mice	Berger 2010	[39]
	Rat	Wister-derived	N.R.	Subcutaneous Injections	PND 1, 3, 5, and 7	0.05 mg/kg/day and 20 mg/kg/day	PND 1, 3, 5, and 7	Pregnancgy rate decrease. Decrease in the number of implantation sites. A lower mRNA expression of Hoxa10 and a lower protein expression of ER and PR.	Adult female rats	Varayoud 2011	[40]
	Mouse	CD-1	PND56	Subcutaneous injections	N.R.	0, 60, 600 mg/kg/ day	N.R.	Downregulation of PGR and HAND2 expression in uterine stroma upon BPA exposure was associated with enhanced activation of FGF and MAPK signaling in the epithelium, contributed to aberrant proliferation and lack of uterine receptivity	N.R.	Li 2016	[98]
	Mouse	Sensitivity to FVB	12 weeks of age	Oral administration	From GD 11 until birth	0.5, 20, and 50 μg/kg	PND 4	Disruption of germ cell nest breakdown and reduce of the size of the primordial follicle pool by altering the expression of pro- and anti-apoptotic factors. Advance of puberty onset and disturb of estrous cyclicity.	Ovaries of female offspring at PND4	Wang 2014	[100]
	Mouse	Sensitivity to FVB	12 weeks of age	Feeding Exposure	From GD 11 until birth	0.5, 20, and 50 μg/kg	On PND 4 and PND 21	BPA at 50 μg/kg/day increased expression of the anti-apoptotic factor Bcl2 and BPA at 0.5 μg/kg/day and 20 μg/kg/day decreased expression of the pro- apoptotic Bax compared to control. In the F2 generation, BPA at 0.5 μg/kg/day significantly decreased expression of Bcl2, but it did not significantly affect the expression of Bax compared to control. BPA also did not significantly affect the ratios of these two factors in the F2 ovaries. In the F3 generation, BPA exposure did not significantly affect levels of expression of Bcl2, Bax, or their ratio compared to control. In utero BPA exposure inhibits germ cell nest breakdown in PND 4 ovaries of the F1 generation, but not in the F2 or F3 generations.	Female offspring in adulthood	Berger 2016	[99]
	Experimental Mouse studies in vivo	Kunming	N.R.	Gavage	From GD0.5 to GD3.5	200, 400, 600, and 800 mg/kg/day	From GD0.5 to GD3.5.	Delay of the transfer of embryos to the uterus, damaged blastocyst development before implantation, and inhibited embryo implantation.	Adult female mice	Pan 2015	[37]
	Mouse	C57BL6	2–3 months old	Subcutaneous Injecton	From GD0.5 to GD3.5	0, 0.025, 0.5, 10, 40, and 100 mg/kg/day	From GD0.5 to GD3.5.	Delayed implantation and increased perinatal lethality of their offspring were observed.	Pregnant female mice	Xiao 2011	[38]
	Mouse	CD-1	N.R.	Subcutaneous Injections	GD9–GD16	0.1, 1, 10, 100, or 1000 μg/kg/day	16–18 months	Ovarian cysts increase; increasing in proliferative lesions of the oviduct; squamous metaplasia of the uterus; some evidence of a rise with the lowest dose and declines with increasing dose.	Female offspring in adulthood	Newbold 2009	[101]
	Rat	Wister-derived	N.R.	Subcutaneous Injections	PND 1, 3, 5, and 7	0.05 mg/kg/day and 20 mg/kg/day	PND 1, 3, 5, and 7	Pregnancgy rate decrease. Decrease in the number of implantation sites. A lower mRNA expression of Hoxa10 and a lower protein expression of ER and PR.	Adult female rats	Varayoud 2011	[40]
	Mouse	CD-1	PND56	Subcutaneous injections	N.R.	0, 60, 600 mg/kg/ day	N.R.	Downregulation of PGR and HAND2 expression in uterine stroma upon BPA exposure was associated with enhanced activation of FGF and MAPK signaling in the epithelium, contributed to aberrant proliferation and lack of uterine receptivity	N.R.	Li 2016	[98]
	Mouse	Sensitivity to FVB	12 weeks of age	Oral administration	From GD 11 until birth	0.5, 20, and 50 μg/kg	PND 4	Disruption of germ cell nest breakdown and reduce of the size of the primordial follicle pool by altering the expression of pro- and anti-apoptotic factors. Advance of puberty onset and disturb of estrous cyclicity.	Ovaries of female offspring at PND4	Wang 2014	[100]
	Mouse	Sensitivity to FVB	12 weeks of age	Feeding Exposure	From GD 11 until birth	0.5, 20, and 50 μg/kg	On PND 4 and PND 21	BPA at 50 μg/kg/day increased expression of the anti-apoptotic factor Bcl2 and BPA at 0.5 μg/kg/day and 20 μg/kg/day decreased expression of the pro- apoptotic Bax compared to control. In the F2 generation, BPA at 0.5 μg/kg/day significantly decreased expression of Bcl2, but it did not significantly affect the expression of Bax compared to control. BPA also did not significantly affect the ratios of these two factors in the F2 ovaries. In the F3 generation, BPA exposure did not significantly affect levels of expression of Bcl2, Bax, or their ratio compared to control. In utero BPA exposure inhibits germ cell nest breakdown in PND 4 ovaries of the F1 generation, but not in the F2 or F3 generations.	Female offspring in adulthood	Berger 2016	[99]
	Experimental Mouse studies in vivo	Kunming	N.R.	Gavage	From GD0.5 to GD3.5	200, 400, 600, and 800 mg/kg/day	From GD0.5 to GD3.5.	Delay of the transfer of embryos to the uterus, damaged blastocyst development before implantation, and inhibited embryo implantation.	Adult female mice	Pan 2015	[37]
	Mouse	C57BL6	2–3 months old	Subcutaneous Injecton	From GD0.5 to GD3.5	0, 0.025, 0.5, 10, 40, and 100 mg/kg/day	From GD0.5 to GD3.5.	Delayed implantation and increased perinatal lethality of their offspring were observed.	Pregnant female mice	Xiao 2011	[38]
	Mouse	CD-1	N.R.	Subcutaneous Injections	GD9–GD16	0.1, 1, 10, 100, or 1000 μg/kg/day	16–18 months	Ovarian cysts increase; increasing in proliferative lesions of the oviduct; squamous metaplasia of the uterus; atypical hyperplasia and stromal polyps of the uterus; sarcoma of the uterine cervix.	Female offspring in adulthood	Newbold 2009	[101]
	Mouse	CD-1	N.R.	Subcutaneous injections	PND 1–5	10, 100, or 1000 μg/kg/day	18 months	Significant increase in cystic ovaries and cystic endometrial hyperplasia; progressive proliferative lesion of the oviduct and cystic mesonephric duct remnants; adenomyosis, leiomyomas, atypical hyperplasia, and stromal polyps of the uterus.	Adult female mice	Newbold 2007	[102]
	Rat	Wistar	N.R.	Drinking water	GD9-PND21	10 mg/L	3 months	Modification of estrous cyclicity, an increased height of both uterine epithelia and stroma, a reduction in apoptotic cells in both uterine luminal and glandular epithelium and down regulation of ER alpha on estrus days.	Female offspring in adulthood	Mendoza- Rodriguez 2011	[103]
	Mouse	CF-1	3–6 months	Subcutaneous injections and Oral administration	From GD1–4	0.000, 0.0005, 0.0015, 0.0046, 0.0143, 0.0416, 0.125, 0.375, 1.125, 3.375, and 10.125 mg BPA/animal/ day	From GD1–4	Subcutaneous injections resulted in a significant decrease in the average number of pups at 3.375 mg/day. At 10.125 mg/day, there was a significant reduction in the number of pregnancies. Uterine implantation sites were also significantly reduced in females sacrificed at day 6 after receiving 10.125 mg/day.	Pregnant female mice	Berger 2007	[104]
	Mouse	CF-1	3–6 months old	Subcutaneous Injections	GD1–4	6.75 and 10.125 mg/mL	From GD1 to GD4	In Experiment 1, daily doses of 6.75 and 10.125 mg significantly reduced the number of implantation sites. Urinary progesterone was significantly reduced by the higher dose. In Experiment 2, inseminated females received a single dose of BPA on days 0, 1, or 2 of gestation. A single dose of 10.125 mg reduced the number of implantation sites when given on day 0 or day 1, and 6.75 mg on day 1 also produced fewer implantation sites.	Pregnant female mice	Berger 2008	[105]
	Mouse	CD-1	Pregnant mice	Oral administration		0.02, 0.04, 0.08 mg/kg	PND3, 5, 7	BPA exposure level was associated with more oocytes in germ cell cyst and less primordial follicle. Decreased mRNA expression of specific meiotic genes including Stra8, Dmc1, Rec8 and Scp3 were observed.	Female offspring in adulthood	Zhang 2012	[106]
	Ewes	Corriedale	2–4 years old	Subcutaneous injections	PND 1–14	5 and 50 μg/kg/ day	PND30	Reduce in ovarian weight and increase in the number of multioocyte follicles. Proliferation of granulosa/theca cells in antral follicles and increase of the number of antral atretic follicles. Reduce in the primordial follicle pool by stimulating their initial recruitment and subsequent follicle development until antral stage. Acceleration of folliculogenesis resulted in increased incidence of atretic follicles.	Female lamb at PND30	Rivera 2011	[107]
	Ewes	Corriedale	2–4 years old	Subcutaneous Injections	PND1–14	0.5, 5 and 50 μg/kg/day	PND30 and PND34	Impaired ovarian response to oFSH with a lower number of follicles. AR induction by oFSH disruption in granulosa and theca cells. An increase in GDF9 mRNA expression levels. In contrast, a decrease in BMPR1B was observed.	Ovaries of female lamb at PND30 and after oFSH at PND34	Rivera 2015	[108]
	Rat	Wistar	90 days old	Drinking water	GD9-PND21	2.5, 50 and 250 μg/ kg/day	PND90 during estrus	Ovaries showed reduced primordial follicle recruitment and a greater number of corpora lutea. A lower expression of androgen receptor (AR) at different stages of the growing follicle population was demonstrated.	Female offspring in adulthood	Santamaria 2016	[109]
	Mouse	CD-1	N.R.	Hypodermical injiection	PND7–14	20 and 40 μg/kg/ day	PND15 and PND 21	BPA promotes the primordial to primary follicle transition, thereby speeding up the depletion of the primordial follicle pool, and suppressed the meiotic maturation of oocytes because of abnormal spindle assembling in meiosis.	Adult female mice	Chao 2012	[110]
	Rat	Sprague- Dawley	28 days- old	Oral gavage	42 days	10 mg/kg bw/day	After the last treatment day, during diestrus phase	Higher number of large antral follicles and atretic follicles that did not reached ovulation stage	The same adult female rats (70-days- old)	Zaid 2018	[88]
	Rat	Wistar	Female pups	Subcutaneous Injection	From PND 1 to PND 15	25 ng/kg/d and 5 mg/kg/d	90 days after last treatment, during diestrus phase	Decreased number of primordial follicles and increased number of atretic follicle at both tested BPA doses	Female offspring in adulthood (PND 105)	Lòpez-Rodríguez 2019	[89]
	Rat	Wistar	Adult female	Subcutaneous Injection	From PND 90 to PND 105	25 ng/kg/d and 5 mg/kg/d	24 h after last treatment, during diestrus phase	Decreased number of antral follicles and increased number of corpora lutea at both tested BPA doses	The same adult female rats (105-days- old)	Lòpez-Rodríguez 2019	[89]

*N.R* not reported

### Experimental studies in vitro and ex vivo

Several experimental in vitro and ex vivo studies have investigated the effects of BPA exposure on ovary and uterus morphology and functions, including ovarian development, folliculogenesis, uterine receptivity and embryo implantation [32, 35–40, 95–100]**.**

Experimental ex vivo studies on ovary confirmed that exposure to BPA affected folliculogenesis, particularly follicle growth and development, by interfering with multiple mechanisms such as proliferation and apoptosis, beyond steroidogenesis, of ovarian cells. The administration of low BPA doses in ovaries explanted from mouse female pups at PND1 or PND4 inhibited germ cell nest breakdown and enhanced primordial follicle recruitment, by decreasing the expression of Ki-67, Fas, Bac, Bax and caspase 3 and 8, increasing the expression of Bcl2, and activating PI3K/Akt pathway [95, 96, 100]. Moreover, in ovarian follicles isolated from preantral to antral stages from female adult mice low and high BPA doses affected different phases of folliculogenesis: in particular, low BPA doses accelerated follicle development with an increase in antral follicle growth [35], while high BPA doses selectively inhibited antral follicle growth [32, 36, 97]. Low BPA doses effects on antral follicles were found to be associated with high methylation level of several maternally and paternally imprinted genes [35], whereas high BPA doses effects on antral follicles were found to be mediated by interference with the expression of genes involved in cell cycle progression (increased expression of cyclin-dependent kinase 4 (Cdk4) and cyclin E1 (Ccne1) and decreased expression of cyclin D2 (Ccnd2)) [36], and apoptosis (increased expression of p53, Bcl-2 and Bax) [36]. Ovaries explanted at PND4 and PND21 from the first (F1) and the third (F3) female generations of mice prenatally and perinatally exposed to low BPA doses and subjected to histological evaluation and gene expression analyses revealed that BPA exposure did not have transgenerational effects on germ cell nest breakdown and gene expression on PND4, but it caused transgenerational changes in expression of multiple steroidogenesis-related genes on PND21, demonstrating a difference in transgenerational transmittance of the BPA effect [99].

Experimental ex vivo studies on uterus confirmed that exposure to BPA affected uterus morphology and function, particularly interfering with uterine receptivity and embryo implantation. An ex vivo study, conducted on uterus of female adult mice treated with BPA in the first 3 days of pregnancy, demonstrated that high BPA doses delayed the transfer of embryos to the uterus, damaged blastocyst development before implantation, and inhibited embryo implantation. Regarding the putative mechanism, high BPA doses exposure induces a dose-dependent increase of endothelial nitric oxide synthase (eNOS) protein expression in trophoblast cells, the cells forming the outer layer of a blastocyst, with consequent induction of NO excess, which might represent one of the causal factor involved in embryo implantation [37]. Moreover, in explanted uterus of female adult mice and rats treated with low and high BPA doses a reduced number or a completed abrogation of implantation sites were found, together with an adversely affected uterine receptivity [37–40]. Unfavorable embryo implantation was also observed in female adult mice exposed to low BPA dose treatment, in which ex vivo analysis of uterus showed reduced decidual cells surrounding the attached embryo, and an increased percentage of intrauterine hemorrhage, due to the shedding and collapse of the endometrium, which make critical the embryo implantation [98]. The impairment of uterine receptivity and the unfavorable embryo implantation can be addressed by the BPA capability to regulate uterine morphology by increasing uterine luminal area and enhancing the uterine luminal epithelial cell height [39], and by the BPA capability to influence uterine functions by affecting E2 and P/HAND2 (heart and neural crest derivatives expressed 2) pathway [98]. Indeed, BPA exposed uterine epithelial and stromal tissues showed a marked suppression of E2 and P receptor protein expression and P receptor downstream target gene, HAND2, in uterine epithelial and stromal uterine cells, consequently enhancing the activation of fibroblast growth factor and MAPK signaling in the epithelium, thus contributing to aberrant proliferation, lack of uterine receptivity and embryo implantation, and reducing the establishment of pregnancy [38–40, 98].

### Experimental studies in vivo

Several experimental in vivo studies have investigated the effects of BPA exposure on ovary and uterus morphology and functions, including ovarian development, folliculogenesis, uterine receptivity and embryo implantation [37, 38, 88, 89, 101–110].

The effect of BPA on ovarian morphology and functions has been investigated in female pups and adult animals during different phases of estrus cycle. Prenatal (from GD9 to 16) and postnatal (from PND1 to PND21) exposure to low BPA doses by oral and subcutaneous administration in mouse and rat female pups induced inhibition of germ cell nest breakdown, a decrease of the number of primordial follicles and an increase of their recruitment, as well as an increase of the number of atretic follicles and of ovarian cysts, assessed during first day of life and in adulthood during estrus and diestrus [89, 101, 106, 109]. Moreover, prenatal and perinatal exposure to low BPA doses has been shown to affect folliculogenesis and ovarian morphology, as assessed in adult offspring of several animal models, including mice, rats and ewes, by inducing the development of multi-oocyte follicles, decreasing follicle type proportion or number and reducing ovarian weight [107–110]. BPA induces ovarian alterations by interfering with multiple molecular processes and pathways involved in folliculogenesis. Indeed, it has been shown that BPA enhances ER messenger expression in ovary and, through the binding to ER, induces epigenetic modifications, in particular DNA hypomethylation, of genes involved in oocyte maturation, with a consequent acceleration of the transformation of the primordial to primary follicle [110]. Impaired folliculogenesis has been also observed in female adult rats exposed to low BPA doses; indeed, a higher number of corpora luteal, large antral-like follicles and atretic follicles, have been observed in BPA treated female adult rats [88, 89]. Interestingly, Lopez-Rodriguez demonstrated that the disruption of folliculogenesis is a reversible process depending on the window of BPA exposure. Indeed, when BPA exposure occurs at adulthood the alterations appear to be transient, as confirmed by the reduced number of antral follicles and corpora lutea and the restoration of physiological condition few weeks later after the end of BPA exposure, whereas disruption of folliculogenesis, represented by decreased number of primordial follicles and increased number of atretic follicles, persists into adulthood following postnatal exposure, indicating a disruption of ovarian programming in dependence of the window of BPA exposure [89].

Experimental in vivo studies in mice demonstrated that both prenatal and perinatal exposure to low BPA doses affected oviduct morphology. In particular, in female offspring mice prenatally exposed to low BPA doses an increased number of progressive proliferative lesions in the oviduct was found at adulthood (16 to 18 months of age) [101]. Consistently, in mouse female pups postnatally treated with low BPA doses, from PND1 to PND5, an increased number of progressive proliferative lesions of the oviduct was found at 18 months of age [102]. Moreover, female pregnant mice treated by gavage and subcutaneous administration of low and high BPA doses in the first 3 days of gestation showed a significant reduction of embryo development and transport throughout the oviduct when treated with high but not with low BPA doses, suggesting that high BPA doses compromise both morphology and functions of the oviduct [37, 38].

Several experimental in vivo studies in rodent models demonstrated that exposure to BPA from prenatal to adult age, at both low and high doses, affected uterus morphology and functions, in particular uterine receptivity. Mice female offspring prenatally exposed to low BPA doses increased the incidence of proliferative lesions of the uterus, including atypical hyperplasia and stromal polyps of the uterus, as well as sarcoma of the uterine cervix, assessed at 16 to 18 months of age [101]. Moreover, rat female offspring, prenatally and postnatally exposed to low BPA doses, showed increased thickness of uterus and stroma epithelia and a reduction in apoptotic cells in both uterine luminal and glandular epithelium assessed at 3 months of age [103]. Moreover, the effect of BPA treatment on the uterine receptivity was also studied on female pregnant mice in which treatment with both low and high BPA doses in the first 3 days of gestation, induced a reduction of implantation sites [37, 38, 104, 105].

### Observational studies in humans

The effect of BPA on ovarian, oviduct, and uterus morphology and functions has not been investigated in humans although several experimental evidences suggest that BPA could have a detrimental impact on the morphology and functions of female reproductive system.

A recent study evaluating the correlation between the urinary BPA levels and ovarian outcomes in a prospective cohort of women undergoing MAR reported that higher urinary BPA levels were associated with lower antral follicle count, raising concern for possible accelerated follicle loss [111]. More epidemiological studies are warranted to confirm the experimental data on the effect of BPA on female reproductive system in humans.

Taken together, these findings demonstrate that exposure to low and high BPA doses has harmful effects on ovarian morphology, in particular in several steps of ovarian development, and functions, in particular folliculogenesis, and on uterus morphology and functions in female adult animal and offspring. The different outcome might likely depend on animal model, BPA doses, administration route and window of exposure. In particular, prenatal, perinatal and postnatal BPA exposure, although with variable results among the studies, to low and high doses mainly regulate: 1) ovarian development, observed in the pups in the first days of birth, reducing the breakdown of germ cell nest, as consequence of deregulated expression of apoptotic genes; 2) oviduct morphology; 3) folliculogenesis, interfering with the growth of antral follicle as consequence of deregulating expression of genes involved in cell cycle and of steroidogenic enzymes, increasing the number of atretic follicles, decreasing the number of corpora lutea and accelerating the transformation of the primordial to primary follicle, this latter mainly due to an ER-dependent hypomethylation of genes involved in oocyte maturation; and 4) uterine morphology, uterine receptivity, and embryo implantation and therefore the establishment of pregnancy. Compromise uterine functions are mainly due to NO excess condition deregulation of P/HAND2 pathway in epithelial and stromal uterine cells.

Importantly, disruption of folliculogenesis has been found to be a reversible process depending on the window of BPA exposure. Indeed, damages induced by BPA exposure in adulthood appear to be transient as confirmed by the restoration of physiological condition after the end of BPA exposure, whereas disruption of folliculogenesis persists into adulthood following postnatal exposure, indicating a disruption of ovarian programming in dependence of the window of BPA exposure.

## Bisphenol and female reproductive disorders

A growing body of evidence have been reported that exposure to BPA may contribute to the pathogenesis of female reproductive disorders, due to its ability to disturb endocrine activity in animals and humans. In particular, most of the evidence have been reported a role of BPA in the pathogenesis of endometriosis, a chronic inflammatory disease where cells similar to endometrial cells are found outside the uterine cavity, most commonly in the pelvis [112]. Furthermore, the female gonad appears to be a particularly sensitive target of BPA, as indicated by evidence of interference with ovarian morphology and ovarian functions, such as steroidogenesis, and particularly folliculogenesis. All these mechanisms might contribute to the pathogenesis of PCOS, a complex condition characterized by increased androgen levels, menstrual irregularities, and/or small cysts on one or both ovaries [113]. The following paragraphs describe the available data on the role of BPA in the pathogenesis of endometriosis and PCOS, obtained in experimental in vitro, ex vivo and in vivo studies.

### BPA and endometriosis

Endometriosis is a chronic inflammatory disease, characterized by implantation and growth of endometrial tissue outside the uterine cavity [112]. Endometriosis is considered one of the most frequent gynecological diseases, affecting 15–20% of women in their reproductive life. The most common form of the disease is represented by pelvic endometriosis that is associated with increased secretion of pro-inflammatory cytokines, neo-angiogenesis, intrinsic anomalies of the refluxed endometrium and impaired function of cell-mediated natural immunity. Recently, endometriosis has also been considered to be an autoimmune disease due to the presence of autoantibodies, and the association with different autoimmune diseases and recurrent immune-mediated abortion [112]. Endometriosis is considered as the major contributor to pelvic pain and subfertility in young women. The subsequent sections describe the current evidence highlighting the potential association between BPA exposure and endometriosis, obtained in experimental in vitro, ex vivo and in vivo studies, and the association between circulating BPA levels and endometriosis in human observational studies.

### Experimental studies in vitro and ex vivo

Few experimental in vitro and ex-vivo studies have investigated the relationship between BPA exposure and the occurrence of endometriosis and it has been reported that BPA exposure increases the incidence of endometriosis-like lesions.

suggesting a hypothetical role of BPA in the pathogenesis of endometriosis [19, 42]. The experimental in vitro, ex vivo and in vivo studies reporting the effects of BPA on uterine endometrium are summarized in Table 5**.** These studies are limited by the fact that they have not been carried out in experimental models of endometriotic cells, but they were performed in endometrial cells exposed to BPA in order to investigate if the exposure to BPA could induce the histological pattern of endometriosis.

**Table 5:** Bisphenol A and endometriosis

	Source	Strain	Age	Exposure route	Time of exposure	Doses	Time of observation	Outcome	Outcome observed in	Reference n°	
Experimental studies in vitro and ex vivo	Mouse	C57Bl/6 N and CD-1 mice	Adult mice	Oral administration	12–15 weeks	0.004, 0.04, 0.4, 4, and 40 mg/kg/day	19–23 weeks of age	C57B1/6 N mice had an increased collagen fiber thickness and density in the periglandular regions of stromal cells whereas CD-1 displayed a higher increase in gland nests and extensive periglandular fibrosis	Adult female	Kendziorski 2015	[19]
	Human	Endometrial tissue; stromal cells	35–49 years old	In vitro administration	24 h; 48 h; 72 h	10-5 M; 10-8 M; 10-11 M	24 h; 48 h;72 h	The percentage of cells in G2/M and G0/G1 phase of cell cycle were increased and decreased, respectively, only after treatment with high BPA doses. Low BPA doses were found to increase the gene expression levels of LEFTY after 48 h of treatment, IGFBP1 after 72 h of treatment, and prolactin after 48 h and 72 h of treatment. Increased secretion of IGFBP1 and prolactin was detected after 48 h of treatment with low BPA doses. The messenger expression levels of MMP3 at low BPA doses and messenger expression levels of MMP9 increased at both low and high BPA doses. TIMP3 messenger expression levels were downregulated only at high doses		Forte 2016	[42]
Experimental studies in vivo	Mouse	C57Bl/6 N and CD-1 mice	Adult female	Oral administration	12–15 weeks	0.004, 0.04, 0.4, 4, and 40 mg/kg/ day	19–23 weeks of age	Increased endometrial gland nest formation and stromal and periglandular collagen accumulation was observed in both CD-1 and C57Bl/6 N mouse strains.	Adult Female mice	Kendziorski 2015	[19]
	Mouse	CD1 mice	Female Pups	Subcutaneous injections	From PND1 to PND5	10, 100, or 1000 μg/kg	18 months of age	Increase in cystic ovaries and cystic endometrial hyperplasia in the BPA-100 group.		Newbold 2007	[102]
	Mouse	Balb-C	Adult pregnant female	Subcutaneous injections	From GD1 until the seventh day after delivery	100 or 1000 μg/kg/ day	21 days after delivery	Increase of of endometriosis-like structures in the adipose tissue surrounding the genital organs. Cystic ovaries, aden omatous hyperplasia with cystic endometrial hyperplasia and atypical hyperplasia. Increased gene expression of both ER and Homeobox A10 (HOXA-10) in the nucleus of BPA- induced lesions.	Adult Female mice	Signorile 2010	[98]

To investigate the role of BPA in the pathogenesis of endometriosis, two female adult mouse strains (C57Bl/6 N and CD-1 mice) were exposed to low BPA doses or 17α-ethinyl estradiol as control, for 12–15 weeks [19]. These two strains have been reported to have a different sensitivity to the actions of BPA, with C57Bl/6 N mice more sensitive than CD-1 mice. BPA exposure resulted in an increased endometrial gland nest formation and stromal and periglandular collagen accumulation in both CD-1 and C57Bl/6 N mouse strains. However, when the two strains treated to BPA were compared, C57B1/6 N mice had an increased collagen fiber thickness and density in the periglandular regions of stromal cells whereas CD-1 displayed a higher increase in gland nests and extensive periglandular fibrosis, thus suggesting that genetic background may confer a different susceptibility to the effect of BPA exposure. Regarding to collagen synthesis, BPA increased messenger expression levels of collagen type I alpha 1 chain (Col1a1), which is the major component of type I collagen, and collagen type III alpha 1 chain (Col3a1), which is one of the fibrillar collagens whose proteins have a long, inflexible, triple-helical domain in BPA exposed mice. However, BPA exposure had the opposite effect on collagen degradation through matrix metalloproteinase (MMP), which are enzymes degrading extracellular matrix proteins. In particular, messenger expression levels of Mmp2, protein expression levels of MMP2 and MMP14 and activity of MMP2 were decreased in BPA exposed mice. This decrease in MMP expression and activity may give rise to decreased collagen degradation thus leading to an accumulation of collagen in the stromal and periglandular compartments of the endometrium. All these *BPA related* changes contribute to the onset of an endometriosis-like phenotype [19].

**Fig. 1:**
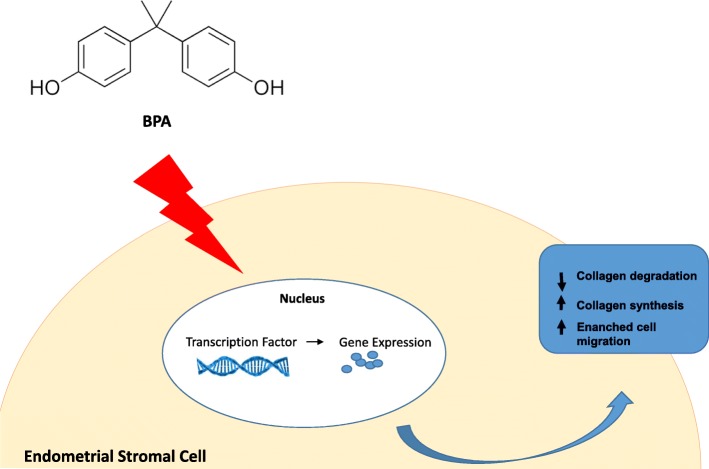
The effect of BPA in the pathogenesis of endometriosis. Endometriosis is classically defined as the presence of endometrial glands and stroma in ectopic locations, primarily the pelvic peritoneum. BPA exposure has been associated with a negative balance of collagen synthesis and an enhanced cell migration

Only one experimental in vitro study investigated the effects of BPA on human endometrial cells; in particular, human endometrial stromal cells (ESCs), isolated from the uterus of healthy subjects during the proliferative phase of the menstrual cycle, were exposed to low and high BPA doses, and the effects on cell cycle progression and decidualization markers gene expression were assessed after 24 h, 48 h, 72 h of treatment [42]. The results of the study demonstrated that the percentage of cells in G2/M and G0/G1 phase of cell cycle were increased and decreased, respectively, only after treatment with high BPA doses. The effect of BPA on decidualization markers expression, analyzed in ESCs, displayed a differential effect on left-right determination factors (LEFTY), which is a member of the transforming growth factor (TGF)-b family of molecules involved in endometrial extracellular matrix remodeling, IGFBP1 and prolactin messenger expression levels. In particular, low BPA doses were found to increase the gene expression levels of LEFTY after 48 h of treatment, IGFBP1 after 72 h of treatment, and prolactin after 48 h and 72 h of treatment. These changes in gene expression levels were also reflected in increased secretion of IGFBP1 and prolactin, after 48 h of treatment with low BPA doses. Lastly, the results of the study also demonstrated that BPA influences metalloproteases (MMP3, MMP9) and metalloproteases inhibitors (TIMP3) expression, increasing the messenger expression levels of MMP3 at low BPA doses and messenger expression levels of MMP9 at both low and high BPA doses. Conversely, TIMP3 messenger expression levels were downregulated only at high doses [42]. Taken together, these data demonstrated that BPA might alter several features of the human endometrium physiology, including cell cycle progression, decidualization markers expression and the migration potential, therefore contributing to the endometriosis-like phenotype, as reported in Fig. 1.

### Experimental studies in vivo

Few experimental in vivo studies have investigated the relationship between BPA exposure and the occurrence of endometriosis and have been reported that BPA exposure increases the incidence of endometriosis-like lesions [19, 102, 114].

In pelvic organs of BALB-C pregnant mice exposed to low BPA doses, an increase of endometriosis-like structures in the adipose tissue surrounding the genital organs has been found [114]. Furthermore, cystic ovaries, adenomatous hyperplasia with cystic endometrial hyperplasia and atypical hyperplasia were frequently detected in treated animals [114]. Immunohistochemistry performed on BPA-induced lesions confirmed the gene expression of both ER and Homeobox A10 (HOXA-10) in the nucleus, by demonstrating the endometrial nature of these structures [114]. The detection of increased BPA levels in the liver of both the pregnant BPA exposed mice and offspring of exposed mice highlighted the hypothesis that BPA is trans-generationally distributed from the mother to the offspring, and strengthen the hypothesis that the prenatal exposure to BPA might be the underlying cause of the observed increase in the occurrence of endometriosis [114]. These findings are in agreement with another study in which an increase of cystic ovaries and of cystic endometrial hyperplasia has been found in CD-1 mice postnatally exposed to low BPA doses [102]. The observed accumulation of BPA in the liver of pregnant mice and offspring confirmed previous evidence that BPA is efficiently absorbed and distributed across generations to several organs, including the reproductive system [115]. Taken together, these findings suggest that BPA exposure could trigger the pathogenic mechanisms involved in endometriosis. In addition, BPA exposure during early lifestage may have a transgenerational effect predisposing the subsequent generations to the risk of developing this disease.

### Observational studies in humans

Several observational studies have been carried out to investigate the possible association between BPA exposure and the occurrence of endometriosis in humans [116–122]. The possible link between serum BPA levels and the occurrence of endometriosis has been firstly assessed by a prospective cross-sectional study in 2007 [116]. In detail, Itoh et al. investigated differences in terms of urinary BPA levels among 140 women aged between 20 and 45 years with different stage of endometriosis according to American Fertility Society classification [116, 117]. Although higher unadjusted urinary BPA levels were observed in advanced endometriosis, no differences between different endometriosis stages emerged. The main bias of this study resides in the absence of a control group without endometriosis and the single measurement of urinary BPA levels that could not account for the long-term exposure. Furthermore, these findings were not confirmed by a subsequent prospective case-control study authored by Cobellis et al. involving 11 healthy women aged 35–39 years and 58 women aged 33–39 years with endometriosis; in this study, urinary BPA levels were detected in 51.7% of 58 patients with endometriosis suggesting possible relationship between endometriosis and BPA [118]. In the same line are the results reported in a prospective case-control study carried out by Simonelli et al. enrolling 60 women with endometriosis and 68 healthy women aged 30–40 years, in which higher urinary BPA levels were observed in women affected by endometriosis [119]. Focusing on ovarian endometriosis, Rashidi et al. in a prospective case-control study on 50 Iranian women with endometrioma and 50 healthy women aged 32–37 years detected higher urinary BPA levels in patients with endometrioma [120]. Conversely, the large prospective case-control study by Upson et al. carried out a prospective case control study in 143 women with endometriosis (75 women with ovarian endometriosis and 68 women with non ovarian pelvic endometriosis) and 287 healthy women aged 28–49 years. Although an association between urinary BPA levels and endometriosis overall was not found, a positive association between urinary BPA levels and non-ovarian endometriosis was detected [121]. Furthermore, Moreira-Fernandez et al. carried out a prospective cross-sectional study in 30 women with endometriosis and 22 women without endometriosis aged 18–45 years failing to find an association between urinary BPA levels and endometriosis diagnosed using video laparoscopy surgery [122]. In conclusion, despite most observational studies in humans observed a relationship between BPA and endometriosis, this is not a consistent evidence and requires further studies to shed light on this issue. Furthermore, BPA exposure might also have a role in the severity of endometriosis as highlighted by higher BPA levels detected in women having advanced endometriosis compared to women having less severe stages of endometriosis.

## Bisphenol A and PCOS

PCOS is a multifactorial metabolic-endocrine disorder characterized by the existence of different phenotypes, affecting women of reproductive age [123]. The diagnosis of PCOS is formulated with the presence of at least two of the three criteria: (1) clinical hyperandrogenism (with hirsutism, acne, seborrhea and alopecia) and/or increased circulating androgens levels; (2) presence of ovarian cysts assessed by ultrasound examination and (3) oligo-amenorrhea with oligo-anovulation, according with the Rotterdam criteria [113]. Growing evidence suggests that BPA exposure may play a role in the pathogenesis of PCOS. The experimental in vitro, ex vivo and in vivo studies reporting the association of BPA with PCOS are summarized in Table 6**.**

**Table 6:** Bisphenol A and PCOS

	Source	Strain	Age	Exposure route	Time of exposure	Doses	Time of observation	Outcome	Outcome observed in	Reference n°	
Experimental studies in vitro and ex vivo	Human	Granulosa-lutein cells	33.8 ± 4.5 years	In vitro administration	72 h	1; 100; 1000; 10,000 ng/mL	72 h	Increase of MMP9 levels at 100 and 1000 ng/mL concentrations. Decrease of MMP9 levels at 10000 ng/mL concentration. Decrease of cell viability at 1000 and 10,000 ng/mL concentrations.		Dominguez 2008	[124]
Experimental studies in vivo	Rats	Sprague-Dawley	Female pups	Subcutaneous injection	From PND1 to PND10	5, 50 and 500 μg/kg bw/day	4–5 months of age	Increase of Testosterone and Estradiol levels and decrease of Progesterone levels in adulthood. Altered GnRH secretion in adulthood. Altered ovarian morphology, increase in cysts number and infertility at 500 μg/kg bw/day. Reduced fertility at 50 μg/kg bw/day. Infertility at 500 μg/kg bw/day.	Adult female	Fernandez 2010	[80]
	Rats	Wistar	Female pups	Drinking water	From GD6 to PND40	1 mg/L	PND40	Induction of PCOS hallmarks.	Pubertal female	Patisaul 2014	[125]

### Experimental studies ex vivo

Only one experimental ex vivo study has investigated the relationship between BPA exposure and the occurrence of PCOS. The wealth of research on PCOS has been provided evidence that genetic, hormonal, metabolic and environmental factors may play a role in the development and clinical manifestations of this complex syndrome. A role of BPA in the pathogenesis of PCOS has been suggested by an experimental ex vivo study in which low BPA doses induced a reduction of cell viability of granulosa-lutein cells isolated from follicular fluid of PCOS women undergoing ART [124]. In the same study, it has been demonstrated that low BPA doses increase the secretion of MMP9 as well as the activity of MMP9 in primary culture of granulosa-lutein cells derived by women with PCOS [124].

### Experimental studies in vivo

Few experimental in vivo studies have investigated the relationship between BPA exposure and the occurrence of PCOS. BPA exposure has been described to influence the secretion of sex hormones affecting ovarian morphology and functions, particularly folliculogenesis with the genesis of PCOS-like abnormalities [80, 125]. Moreover, even perinatal exposure to low BPA doses could promote the development of PCOS-like abnormalities at adulthood [80, 125]. An in vivo study in rats evaluated the differential effects of exposure to phytoestrogens or low BPA doses from prenatal to pubertal phase, on the induction of PCOS hallmarks, including cystic follicles, irregular estrus, elevated body weight and baseline serum glucose, at adulthood. The results of the study showed that either BPA, phytoestrogens, or combined treatments postnatally increased body weight. In adulthood body weight in animal exposed to low BPA doses was higher compared to animal exposed to combined BPA plus phytoestrogens. These results suggest that phytoestrogens mitigated the BPA-induced weight gain [125]. Moreover, BPA treatment induced premature vaginal opening, a sign of puberty, which was hypothesized to be mediated by BPA related weight gain [125]. Rats exposed to phytoestrogens had a higher number of corpus luteum and multiple follicles compared to rats exposed to low BPA doses, which is suggestive of an early ovulation, despite longer estrous cycles [125]; it is unclear whether these changes in ovarian morphology could be translated to a potential younger age at first pregnancy, or to premature follicular depletion [125]. Moreover, rats exposed to phytoestrogens had more cysts at adulthood compared to rats exposed to low BPA doses, confirming a major effect exerted by phytoestrogens comparing with BPA exposure on ovarian morphology [125]. Lastly, phytoestrogens, but not BPA, exposure affected baseline glucose levels [125]. It is noteworthy that circulating androgens levels at adulthood was not changed by exposure of BPA or phytoestrogens [125]. These data are insufficient to discriminate whether BPA might specifically enhance adiposity or overall growth and development [125]. In summary this study highlights the role of BPA in inducing obesity that could be considered as a PCOS related metabolic comorbidity.

### Observational studies in humans

**Fig. 2:**
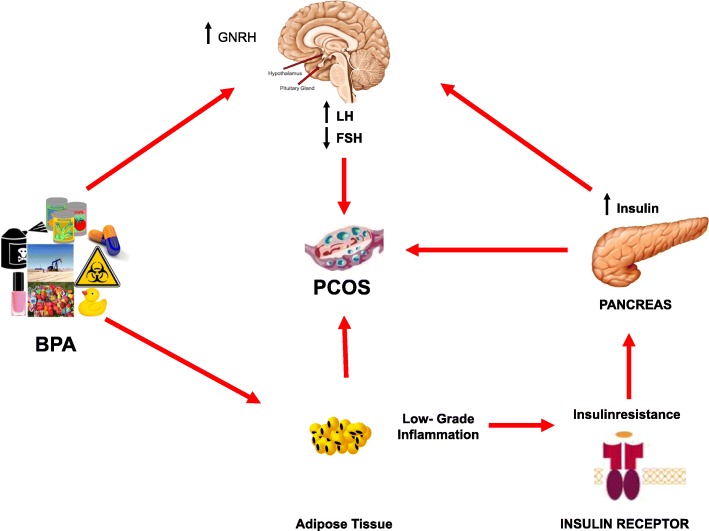
The effect of BPA in the pathogenesis of PCOS. BPA may play a part in the development of PCOS through its steroid potential that can blunt hypothalamic-pituitary-ovarian axis functions by disrupting the steroidal feedbacks at the hypothalamus and pituitary level and steroid action at the level of the ovary. Further, BPA promotes an inflammatory milieu through adipose tissue having a direct action on adipocytes and macrophages infiltrating the adipose tissue and thus contributing to the onset of insulin resistance and compensatory hyperinsulinemia. In turn insulin worsens amplitude and frequency of GnRH and LH pulse secretion seen in PCOS

A hypothetical relationship between PCOS and BPA has drawn attention in recent years and has been suggested by observational studies, although the underlying mechanisms are poorly understood [126–129, 132–135]. BPA has been reported to have a steroid potential thus it can blunt HPO axis functions by disrupting the steroid feedbacks at the hypothalamus and pituitary level and steroid action at the level of the ovary [126]. In addition, BPA seems to contribute to derange metabolic profile in PCOS as reported by a recent metanalysis including 11 case-control studies (seven studies were conducted in Asia and four studies recruited Caucasian participants) and involving 493 PCOS patients and 440 controls in which serum BPA levels were higher in Caucasian PCOS patients, in non-PCOS obese patients and in non-PCOS insulin-resistant patients, therefore leading to hypothesize that BPA might be involved in the insulin resistance of PCOS [127]. In fact, BPA promotes an inflammatory milieu through the development of obesity having a direct action on adipocytes and macrophages infiltrating the adipose tissue [128]. As well-known and showed in Fig. 2, chronic inflammation contributes to the pathogenesis of insulin resistance and compensatory hyperinsulinemia that in turn plays a role in the development of the typical increased amplitude and frequency of GnRH and LH pulse secretion seen in PCOS [129–131]. The fact that BPA was associated with a decrease of antral follicle count in infertile women with PCOS suggests that BPA may impair ovarian reserve [132]. Furthermore, BPA appears to lead to androgen excess, also in lean phenotype, as reported by a prospective observational case control study performed in 112 adolescents with PCOS and 61 controls that had higher serum BPA levels, independently of obesity [134]. This finding has been also found in a prospective observational cross-sectional study carried out in healthy women, where a positive correlation between increased serum T and serum BPA levels has been found [133]. This could be due to the effect of BPA in displacing sex steroid hormones from SHBG therefore increasing the amount of free T. At the same time, it appears that metabolism and excretion of BPA may be impaired in PCOS [135]. This impairment might depend on the effect of androgen excess in blocking the activity and transcription of the liver enzyme uridine diphosphate-glucuronosyl transferase, which clears BPA from circulation under normal conditions; this might partly explain why BPA levels are typically higher in women with PCOS [135]. Taken together these results suggest that BPA levels are higher in women with PCOS than in reproductively healthy women, but the direction of causality has not been established. BPA seems to act disrupting hormonal patterns but also disrupting normal metabolic activity, contributing the to development of PCOS.

## Conclusions

The evidence summarized in the current review suggest that BPA might have a role in the pathogenesis of female infertility. BPA has been often detected in infertile women, thus leading to hypothesize that BPA could interfere with natural conception. BPA exposure has been also associated with negative outcomes of ART and in particular to a decreased E2 production during gonadotropin stimulation, to a decreased number of oocytes retrieved at the end of ovarian stimulation and implantation failure. Findings of studies conducted on animal models pointed out that the deleterious effect of BPA could vary depending on doses, administration route, window of exposure and animal models. In particular, studies on rodent models have demonstrated that the exposure to low BPA doses, but also the high BPA doses, during prenatal and perinatal period, therefore during embryo development, cause dysregulation in HPO axis function and morphological damages to HPO axis organs. Moreover, the BPA exposure in adulthood induces reversible damage in HPO axis function whereas prenatal and perinatal BPA exposure induces irreversible effects in female offsprings. Higher BPA levels have been detected in women with endometriosis and a hypothetical role of BPA in the pathogenesis of endometriosis has been confirmed by experimental animal studies reporting the occurrence of endometriosis-like lesions after BPA exposure. The onset of PCOS-like abnormalities has been found after BPA exposure and this might be induced through the BPA related impairment of the secretion of sex hormones affecting ovarian morphology and functions, particularly folliculogenesis. The several limits of the reported studies prevent to draw final conclusions. This could be due to the fact that most of the studies were retrospective and were not designed aiming to specifically investigate the association between BPA and female fertility. Further, they provide a proxy evaluation of BPA that could not be a parameter to assess the chronic exposure. Therefore, it will be mandatory to perform further studies to investigate the link between BPA and female fertility in order to make the population aware about the health risks related to BPA exposure.

## Data Availability

Literature search results are available from the authors on reasonable request.
